# An Easy and Quick Risk-Stratified Early Forewarning Model for Septic Shock in the Intensive Care Unit: Development, Validation, and Interpretation Study

**DOI:** 10.2196/58779

**Published:** 2025-02-06

**Authors:** Guanghao Liu, Shixiang Zheng, Jun He, Zi-Mei Zhang, Ruoqiong Wu, Yingying Yu, Hao Fu, Li Han, Haibo Zhu, Yichang Xu, Huaguo Shao, Haidan Yan, Ting Chen, Xiaopei Shen

**Affiliations:** 1 Department of Bioinformatics School of Medical Technology and Engineering Fujian Medical University Fuzhou China; 2 Fujian Key Laboratory of Medical Bioinformatics Institute of Precision Medicine Fujian Medical University Fuzhou China; 3 School of Basic Medical Sciences Fujian Medical University Fuzhou China; 4 Department of Critical Care Medicine Union Hospital of Fujian Medical University Fuzhou China; 5 Key Laboratory of Gastrointestinal Cancer (Fujian Medical University) Ministry of Education Fuzhou China; 6 School of Biology and Biological Engineering South China University of Technology Guangzhou China; 7 School of Medical Imaging Fujian Medical University Fuzhou China; 8 Department of Computer Science and Technology, Institute of Artificial Intelligence, Beijing National Research Center for Information Science and Technology Tsinghua University Beijing China

**Keywords:** septic shock, early forewarning, risk stratification, machine learning

## Abstract

**Background:**

Septic shock (SS) is a syndrome with high mortality. Early forewarning and diagnosis of SS, which are critical in reducing mortality, are still challenging in clinical management.

**Objective:**

We propose a simple and fast risk-stratified forewarning model for SS to help physicians recognize patients in time. Moreover, further insights can be gained from the application of the model to improve our understanding of SS.

**Methods:**

A total of 5125 patients with sepsis from the Medical Information Mart for Intensive Care-IV (MIMIC-IV) database were divided into training, validation, and test sets. In addition, 2180 patients with sepsis from the eICU Collaborative Research Database (eICU) were used as an external validation set. We developed a simplified risk-stratified early forewarning model for SS based on the weight of evidence and logistic regression, which was compared with multi-feature complex models, and clinical characteristics among risk groups were evaluated.

**Results:**

Using only vital signs and rapid arterial blood gas test features according to feature importance, we constructed the Septic Shock Risk Predictor (SORP), with an area under the curve (AUC) of 0.9458 in the test set, which is only slightly lower than that of the optimal multi-feature complex model (0.9651). A median forewarning time of 13 hours was calculated for SS patients. 4 distinct risk groups (high, medium, low, and ultralow) were identified by the SORP 6 hours before onset, and the incidence rates of SS in the 4 risk groups in the postonset interval were 88.6% (433/489), 34.5% (262/760), 2.5% (67/2707), and 0.3% (4/1301), respectively. The severity increased significantly with increasing risk in both clinical features and survival. Clustering analysis demonstrated a high similarity of pathophysiological characteristics between the high-risk patients without SS diagnosis (NS_HR) and the SS patients, while a significantly worse overall survival was shown in NS_HR patients. On further exploring the characteristics of the treatment and comorbidities of the NS_HR group, these patients demonstrated a significantly higher incidence of mean blood pressure <65 mmHg, significantly lower vasopressor use and infused volume, and more severe renal dysfunction. The above findings were further validated by multicenter eICU data.

**Conclusions:**

The SORP demonstrated accurate forewarning and a reliable risk stratification capability. Among patients forewarned as high risk, similar pathophysiological phenotypes and high mortality were observed in both those subsequently diagnosed as having SS and those without such a diagnosis. NS_HR patients, overlooked by the Sepsis-3 definition, may provide further insights into the pathophysiological processes of SS onset and help to complement its diagnosis and precise management. The importance of precise fluid resuscitation management in SS patients with renal dysfunction is further highlighted. For convenience, an online service for the SORP has been provided.

## Introduction

Sepsis is a syndrome or group of symptoms associated with infection rather than a single disease [1]. Reportedly, approximately 48**.**9 million cases of sepsis occurred globally in 2017, with approximately 11 million sepsis-related deaths, accounting for approximately 19**.**7% of all deaths worldwide [2]. According to the Third International Consensus on Sepsis and Septic Shock held in 2016 [1], sepsis is defined as life-threatening organ dysfunction caused by a dysregulated host response to infection, and septic shock (SS) is defined as an advanced state of sepsis in which severe circulatory, cellular, and metabolic abnormalities lead to a greater risk of death than sepsis alone, with a mortality rate estimated to be as high as 45% [3,4]. Kumar et al [5] found that patients with SS treated within the first hour of diagnosis had a 79**.**9% survival rate, but each hour of delay in treatment was associated with a 7**.**6% increase in mortality. Therefore, early forewarning of the onset of SS and timely treatment can help reduce the morbidity, mortality, and length of intensive care unit (ICU) stay. However, early forewarning is challenging due to the complexity of the disease in the clinical context [6,7] and the heterogeneity of the sepsis population [8].

Recently, machine learning techniques have been applied to electronic health record data to develop models for risk prediction [9,10], clinical phenotyping [11-13], and early forewarning [14-19]. While several studies have focused on early forewarning of SS in the ICU, practical limitations remain [20-24]. Many models required extensive clinical features from multiple examinations [21,22], increasing the complexity and time for data collection, which can delay forewarnings. Efforts to reduce the feature number often relied on distributed examinations [23] and left limited time for intervention [24]. Additionally, many studies prioritized predictive performance [25-27] while neglecting clinical interpretability. Specifically, limited attention has been given to the triggers of inconsistent predictions, a crucial factor for improving models and the understanding of disease diagnostic criteria. To enhance clinical applicability, models must offer straightforward actionable insights rather than complex algorithms that burden clinicians.

Therefore, we developed a real-time risk-stratified forewarning model for SS that minimized the need for extensive clinical examinations, enabling physicians to quickly and easily assess the patient’s risk and facilitate timely intervention. Furthermore, after stratifying patients based on the risk scores provided by the model, we further analyzed the clinical characteristics of patients in different risk groups, with a particular focus on high-risk (HR) patients. A public web application was deployed that enables easy access and use of our proposed model [28].

## Methods

### Datasets and Participants

To improve the quality and reproducibility of reporting, we prepared a checklist (Multimedia Appendix 1) according to the TRIPOD (Transparent Reporting of a Multivariable Prediction Model for Individual Prognosis or Diagnosis) guidelines [29].

Data were collected from an open-source dataset released for sizeable critical care databases, including the Medical Information Mart for Intensive Care-IV (MIMIC-IV) [30] and the eICU Collaborative Research Database (eICU) [31]. The MIMIC-IV contains comprehensive and high-quality data on deidentified patients admitted to ICUs at the Beth Israel Deaconess Medical Center (BIDMC), with more than 76,000 admissions between 2008 and 2019. The eICU is a multicenter publicly available database containing deidentified highly granular medical data for more than 200,000 patients in 335 ICUs from 208 hospitals across the United States between 2014 and 2015. Both databases include vital signs (VSs), laboratory results, diagnosis, treatment, care plan documentation, severity of illness measures, and other information.

Patients aged 18 years or older who were admitted for the first time and stayed in the ICU for more than 24 hours were included in the analysis. To reduce the impact of complicating factors in defining SS, patients designated as “do not resuscitate” or “encounter for palliative care” according to the International Classification of Diseases (ICD) were excluded. Moreover, patients in the coronary care unit were excluded to reduce the impact of cardiogenic shock. A final cohort of 5125 patients (including 766 SS patients) in the MIMIC-IV and 2180 patients (480 SS patients) in the eICU was obtained. The MIMIC-IV data were randomly partitioned into training (60%), validation (20%), and testing (20%) sets. The eICU data were used for external validation.

### Ethical Considerations

The MIMIC-IV and eICU databases are publicly accessible, deidentified, and transformed, and have been approved for use by the institutional review boards at BIDMC and the Massachusetts Institute of Technology, in accordance with the Declaration of Helsinki. Both databases received waivers for informed consent due to the deidentification of all protected health information. Access to these databases was granted upon completion of human research ethics training and signing of a data use agreement with PhysioNet (certification number 38539898).

### Definitions of Sepsis and SS

The Third International Consensus Definitions for Sepsis and Septic Shock (Sepsis-3) [1] were applied to determine the onset time of sepsis and SS. Specifically, all episodes of suspected infection (t_suspicion_) were identified by the timestamp of antibiotic administration or blood culture, whichever was earlier, within a specific period. If the antibiotic was administered first, the culture sampling was required to be obtained within 24 hours. If the culture sampling occurred first, the antibiotic was required to be ordered within 72 hours. The onset time of sepsis (t_sepsis_) was defined as an episode of suspected infection with a change in the Sequential Organ Failure Assessment (SOFA) score of 2 or more points from up to 48 hours before to up to 24 hours after t_suspicion_. Patients with SS were identified by a clinical construct of sepsis with persisting hypotension requiring vasopressors to maintain mean arterial pressure ≥65 mmHg and with a serum lactate level >2 mmol/L (18 mg/dL) despite adequate volume resuscitation [1]. Specifically, adequate fluid resuscitation was defined as a total fluid volume greater than 30 mL/kg or 1000 mL within a 3-hour window before the initiation of vasoactive drugs or hypotension. The first lactate level needed to be greater than 2 mmol/L within 6 hours after adequate resuscitation. The onset time of SS (t_septic shock_) was the earliest time when all criteria for SS were met. Due to a lack of information on the starting and ending times of fluid resuscitation, SS, according to Sepsis-3, could not be defined in the eICU data. Instead, we used the ICD code labels in the “diagnosis.csv” table to determine SS onset.

### Data Processing

Given the characteristics of time-series data and the need for real-time monitoring for SS risk, this study defined a 6-hour retrospective window, whereby data collected during the 6 hours prior to the current state of the patient were used to provide a forewarning of the SS risk in the next 6 hours. We discretized the features of high-frequency detection, such as VSs, and the features of low-frequency testing, such as laboratory test data, to an hourly value. If multiple measurements were performed in the same hour, their mean value was calculated as the value for this hour of the feature. The forward-filling method was used to fill in missing measurements (with the early measurement), if there was no measurement in this hour.

Next, several time intervals were defined to assess and compare differences among risk groups (derived from the Septic Shock Risk Predictor [SORP] risk scores) in terms of patient pathophysiology (based on VSs and laboratory testing features), clinical characteristics, etc. The time of SS onset is defined as 0. Considering this, –1 indicates 1 hour before onset and +1 indicates 1 hour after onset, and the risk prediction time is –6. Three primary time intervals were delimited, namely, –12 to –6, –6 to 0, and 0 to +6, representing the look-back interval, the preonset interval, and the postonset interval, respectively, where the look-back interval was the time interval used for data collection for risk calculation by the model. For easier comparison among risk groups, the mean value of a clinical feature was used to represent its value within a specified time interval.

Notably, information on patient comorbidities (eg, chronic kidney disease [CKD]) was taken from ICD codes, and invasive procedures (eg, dialysis-continuous renal replacement therapy [CRRT]) were taken from the procedures documented (“procedureevents” table).

### Risk Model

**Figure 1:**
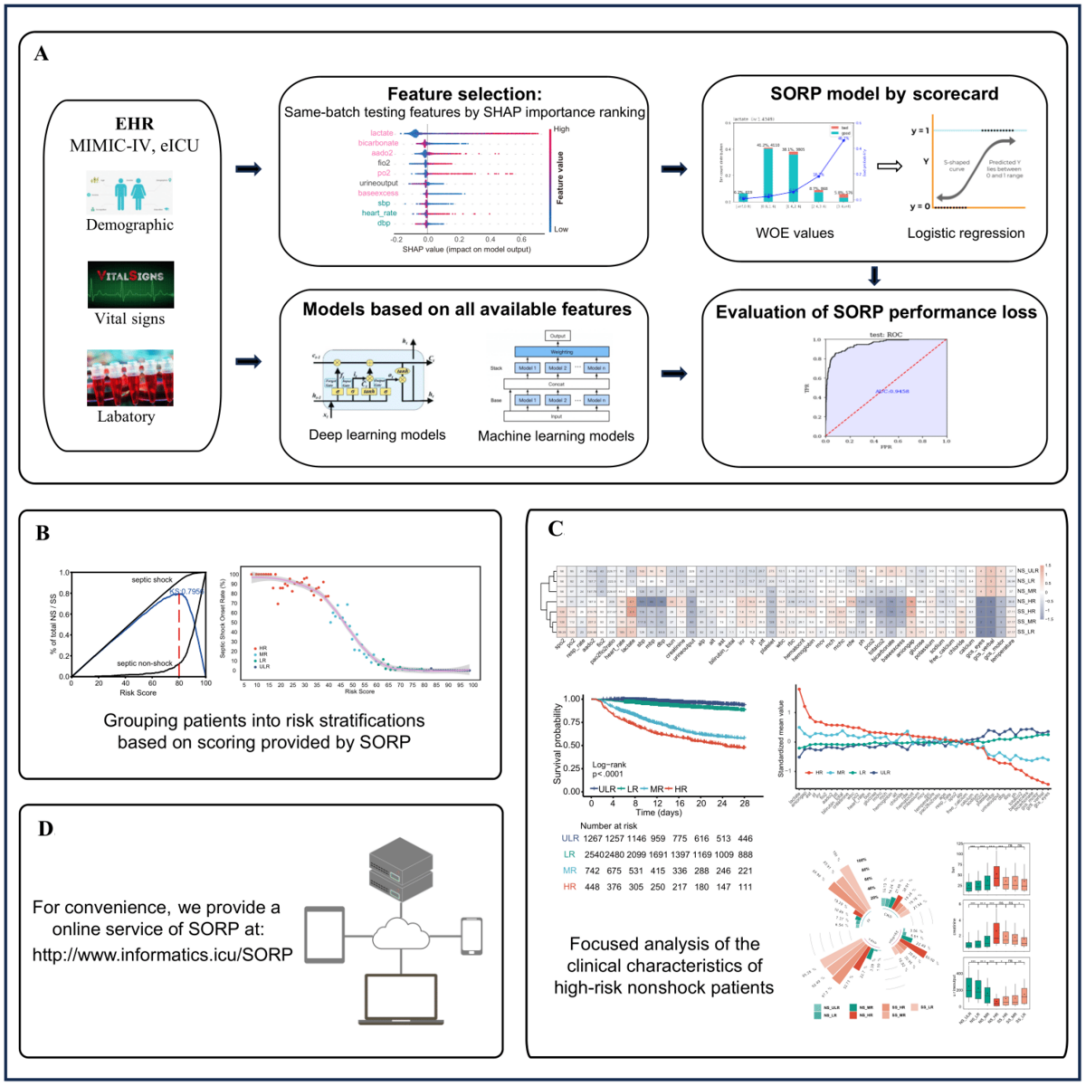
The flowchart of this study. The study flowchart comprises 4 stages. (A) Model development: Electronic health record (EHR) data were collected from the Medical Information Mart for Intensive Care-IV (MIMIC-IV) and eICU Collaborative Research Database (eICU), and multiple forewarning models were constructed. Using Shapley Additive Explanations (SHAP) analysis, we identified key features within the same test batch, which were then used to build the Septic Shock Risk Predictor (SORP) model. The performance of the SORP was compared to that of models based on all available features to evaluate whether the SORP performance loss is acceptable. (B) Risk stratification of septic shock (SS): Patients were stratified into risk groups based on SORP scores. (C) Clinical characteristics of the risk groups: We analyzed clinical characteristics across risk groups, with a focused analysis on high-risk nonshock patients. (D) Model deployment: An online tool was developed for clinical use. HR: high risk; LR: low risk; MR: medium risk; ULR: ultralow risk; WOE: weight of evidence.

To facilitate clinical application, we filtered the features and then developed a scorecard [9,10] to evaluate SS onset risk by logistic regression (LogR). Next, to present the patient’s risk more visually, we stratified the risk scoring and assessed the characteristics of patients in each risk group. The workflow is shown in Figure 1.

The scorecard was constructed as follows. The scorecard allowed for transferring continuous variables into multiple bins, and each feature was divided into multiple bins. In this study, we used the weight of evidence (WOE) to measure the probability of illness in each bin and determine the most appropriate number of bins by detecting the change in information value (IV) under each bin. The WOE and IV were calculated according to equations 1 and 2, respectively. Specifically, first, features were divided into 10 bins, making sure that each bin contained both SS patients and nonshock (NS) patients. Subsequently, the chi-square test was performed on 2 neighboring bins, traversing and merging those bins with the largest *P*-value until the number of bins met the expectation. Then, the final number of bins was determined based on the IV. After the binning was completed, the WOE value of each bin was calculated, and then, the WOE was mapped back to the original dataset to build the LogR model.



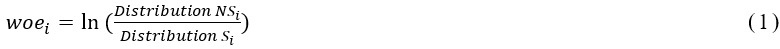





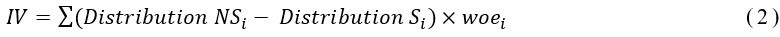



where *i* represents each subgroup, *Distribution* *NS_i_* denotes the proportion of septic NS patients in group *i* to the NS patients in the entire feature, and *Distribution S_i_* denotes the proportion of SS patients in group *i* to the SS patients in the entire feature.

The scorecard points were calculated using the following equations:



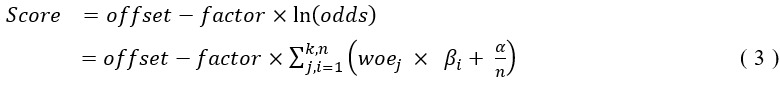





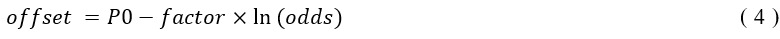





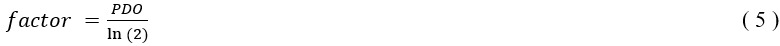



where *P0* and *PDO* (points to double odds) are the score ranges that were set manually, *woe_j_* can be calculated with equation 1, *β_i_* denotes the coefficient of the *i*th feature in LogR, α is the intercept term from LogR, *n* is the number of features, and *k* is the number of bins in each feature.

The scorecard was adjusted to a range of scores between 0 and 100. The final scorecard consisted of a base score and a score for each bin in each feature (Multimedia Appendix 2).

### Statistical Methods

We employed the Shapley Additive Explanations (SHAP) [32] method to calculate the importance of clinical features for SS in the look-back interval. SHAP assigns a value to each feature in the given prediction. These values are calculated for each prediction separately and do not provide general information about the entire model. Higher absolute SHAP values indicate higher importance, whereas values close to 0 indicate low significance of a feature.

To facilitate observation and evaluation and determine risk intervals, we employed the Kolmogorov-Smirnov (KS) curve to describe the overall score distribution. The KS curve illustrated the changes in the sample proportion of SS patients, the proportion of septic NS patients, and the trend of differences between SS and NS patients (blue line) with a score from 0 to 100. From the KS curve, it could be seen that the proportion of SS patients in the low score accumulated faster, while the proportion of NS patients in the high score accumulated faster. The blue curve showed the variation process of the difference between SS and NS patients at each score point. At the highest point of the blue curve, the ability to distinguish between SS and NS patients was the strongest. Therefore, to provide a more direct scoring effect, based on the distributions described above, the scores were divided into 4 risk groups: HR, medium risk (MR), low risk (LR), and ultralow risk (ULR), with score intervals of 0-40, 40-60, 60-80, and 80-100, respectively.

The Population Stability Index (PSI) was used to evaluate the stability of risk group distribution across different datasets in the postonset interval. A smaller PSI value indicates a minor difference between 2 distributions and more consistency. In addition, the area under the curve (AUC) was calculated to evaluate the performance of each model. Estimation and comparison of the survival curves among risk groups were performed using Kaplan-Meier survival analysis and log-rank tests, respectively. The Local Polynomial Regression Fitting (locally estimated scatterplot smoothing [LOESS]) method was used to fit the relationship between the risk score and SS onset rates at 6 hours. Hierarchical clustering was used to assess the similarity between risk groups. All statistical analyses and algorithm approaches in this study were performed using Python (Python Software Foundation) and R (R Project for Statistical Computing).

## Results

### Development of the SORP

**Figure 2:**
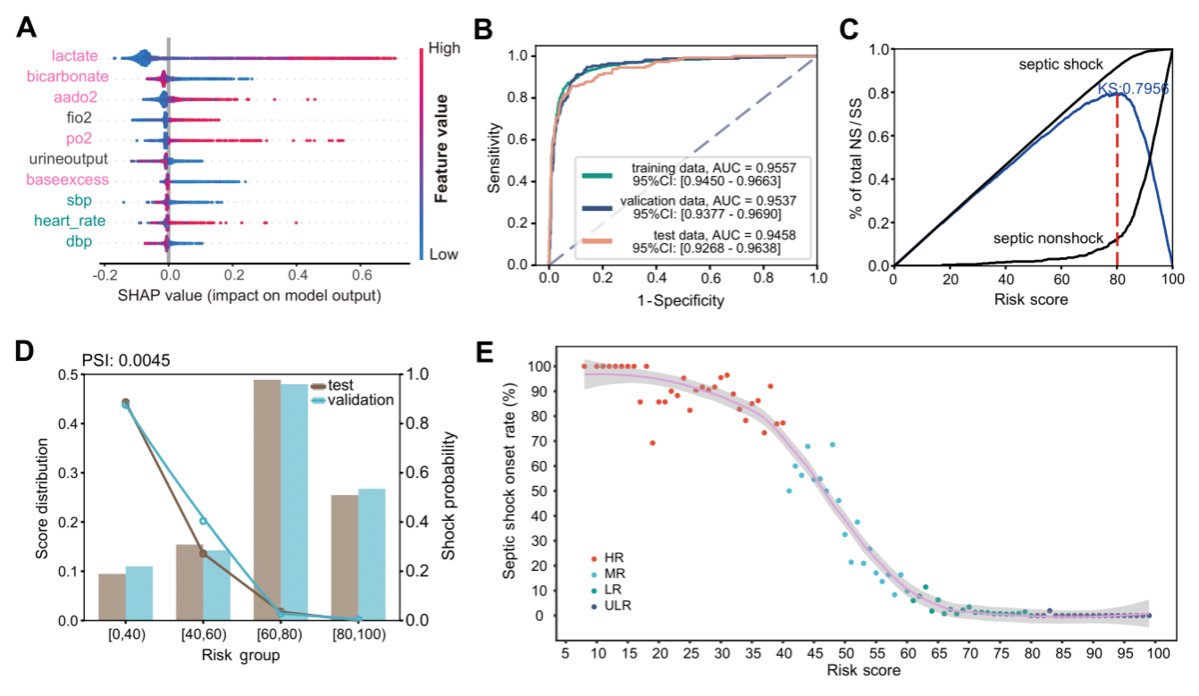
Construction of the Septic Shock Risk Predictor (SORP) and derivation of patient risk stratification. (A) Shapley Additive Explanations (SHAP) summary plot for the top 10 clinical features contributing to the XGBoost model. The position on the y-axis is determined by the feature, in which “hotpink” represents blood gases and “cyan” represents vital signs, and that on the x-axis is determined by the Shapley value. The color from blue to red represents the feature values from low to high. (B) Receiver operating characteristic curve and area under the curve (AUC) for SORP forewarning performance. (C) Kolmogorov-Smirnov (KS) curve shows the difference between the cumulative proportion of septic shock (SS) patients and the cumulative proportion of septic nonshock (NS) patients as the risk score increases (blue line). (D) Stability of SS distribution across datasets. (E) A fitted curve showing the monotonic relationship between the risk score and SS probability in the postonset interval, according to the locally estimated scatterplot smoothing (LOESS) method. HR: high risk; LR: low risk; MR: medium risk; PSI: population stability index; ULR: ultralow risk.

Since a particular time window is required for early forewarning of SS, the longer it takes to collect the necessary data for the model, the less time is left for physicians to intervene. Therefore, we calculated the importance of each feature in the early forewarning of SS in the look-back interval and selected the top-ranked feature set that could be accessed in the same testing batch. In particular, we observed that 8 of the 10 most important clinical features for SS prediction were arterial blood gases (ABGs) and VSs (Figure 2A). Notably, point-of-care ABG testing results could be obtained even within 5-10 minutes in the ICU, and VS features could be obtained at any time from the monitor. This result indicated that septic preshock core pathophysiological shifts could be mainly monitored by ABG and VS features, allowing us to quickly obtain essential features for early forewarning of SS and providing a prompt assessment.

Since features obtained simultaneously from an ABG test might have a certain degree of complementarity, we included 8 ABG and 7 VS features collected in the MIMIC-IV and eICU in the subsequent analysis. An SS risk predictor (SORP) based on a scorecard was constructed by combining LogR with WOE in the look-back interval (detailed in the Methods section). According to the scorecard, a patient’s SS risk score could be obtained by adding interval scores to the base score (Multimedia Appendix 2). Lower total scores indicated a higher risk of SS onset. In the training, validation, and test datasets, the AUCs were 0**.**9557, 0**.**9537, and 0**.**9458, respectively (Figure 2B). Meanwhile, based on all available clinical features, multi-feature complex models were constructed, including deep learning and machine learning models (Figure 1A; Multimedia Appendix 3). These features are related to acid-base balance, coagulation, renal aspects, hepatic aspects, pulmonary aspects, hematologic aspects, the Glasgow Coma Scale, etc. The predictive performance of the SORP is only slightly lower than that of the optimal complex model. In the test datasets, the AUCs of the SORP and the optimal complex model were 0.9458 and 0.9651, respectively. It indicated that the construction of the SORP was reasonable and had strong early forewarning ability.

To facilitate observation and evaluation and determine risk intervals, according to the KS curve plot (Figure 2C), the scores were divided into 4 risk groups as detailed in the Statistical Methods subsection. The distribution of SS was highly consistent and stable among the training, validation, and test datasets (PSI <0**.**01; Figure 2D; Multimedia Appendix 4). In total, the incidences of SS for the HR, MR, LR, and ULR groups in the postonset interval were 88**.**6% (433/489), 34**.**5% (262/760), 2**.**5% (67/2707), and 0**.**3% (4/1301), respectively (Figure 2D; Multimedia Appendix 5). A total of 88**.**6% (433/489) of HR patients developed SS, and 90**.**7% (695/766) of SS patients were classified into the HR or MR group. Further, a curve was fitted for the relationship between the risk score and SS onset rate at 6 hours (Figure 2E; Multimedia Appendix 6), and the result showed a clear stratification and monotonicity, with an increase in the SS onset rate with increasing risk (decreasing risk score). These results suggest that the SORP exhibits a great early forewarning performance.

### Characteristics of Different Risk Groups After SS Onset

**Figure 3:**
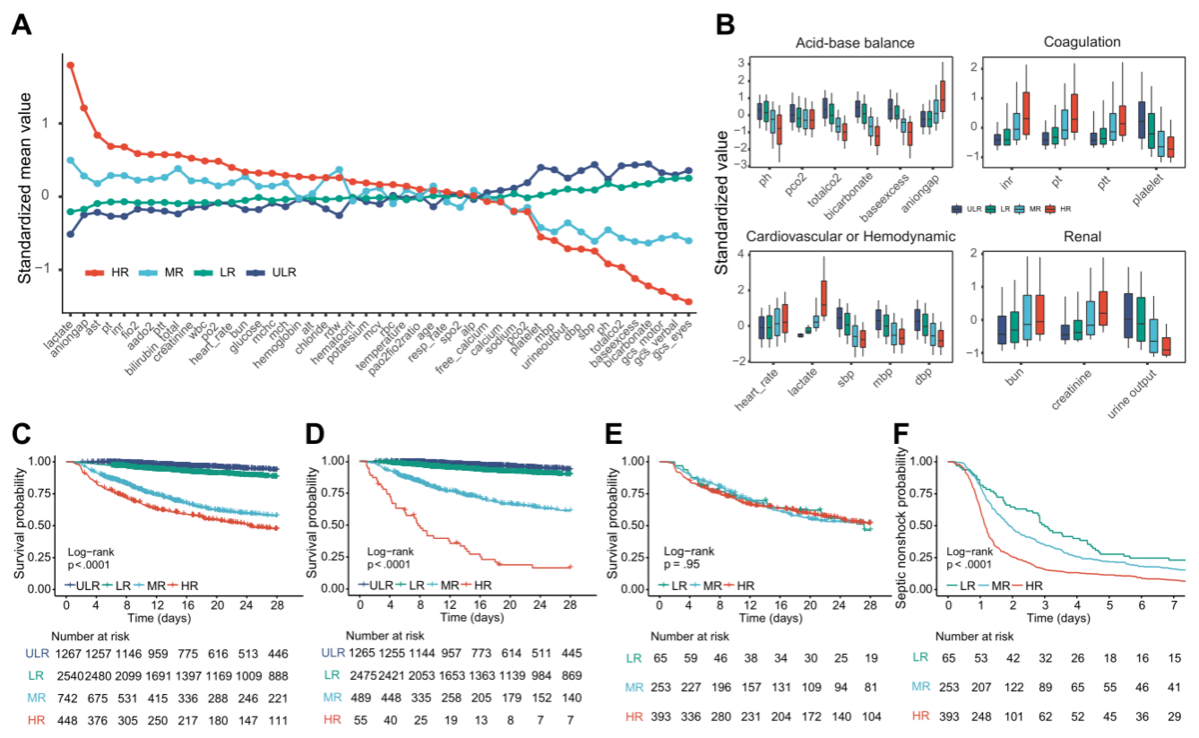
Characteristics of different risk groups in the postonset interval. (A) The clinical features are standardized such that all means are scaled to 0 and SDs to 1. A value of 1 for the standardized mean value (y-axis) signifies that the mean value for the risk group was 1 SD higher than the mean value for the 4 risk groups shown in the graph as a whole. (B) Boxplot showing the changing trend for each clinical feature with risk groups. The y-axis shows the standardized value for each clinical feature. (C-F) Kaplan-Meier curves and survival analysis. (C) Overall survival for all patients. (D) Overall survival for septic nonshock patients. (E) Overall survival for septic shock patients in different risk groups. (F) Time from sepsis to septic shock. HR: high risk; LR: low risk; MR: medium risk; ULR: ultralow risk.

To verify the rationality of risk stratification, we explored the differences in clinical features among risk groups in the postonset interval. Remarkably, increased abnormal values for standardized means of ranking continuous features, especially features related to the SS definition (such as lactate and blood pressure), kidneys, acid-base balance, and coagulation (Figure 3A and 3B; Multimedia Appendix 7), were observed in HR patients when compared with those in other risk groups. The degree of abnormality of these features further increased as risk increased (Figure 3A). For all patients, Kaplan-Meier survival curves progressively declined as patient risk increased (Figure 3C). An even more rapid progressive decline was observed in NS patients in the HR group (Figure 3D), but there was no difference for SS patients (Figure 3E). Furthermore, the length of time from sepsis to SS was significantly reduced with increasing risk (Figure 3F). These results suggest that disease severity increased with increasing risk groups and that the SORP has a reliable risk stratification capability.

### Are NS_HR Patients Truly Septic NS Patients?

**Figure 4:**
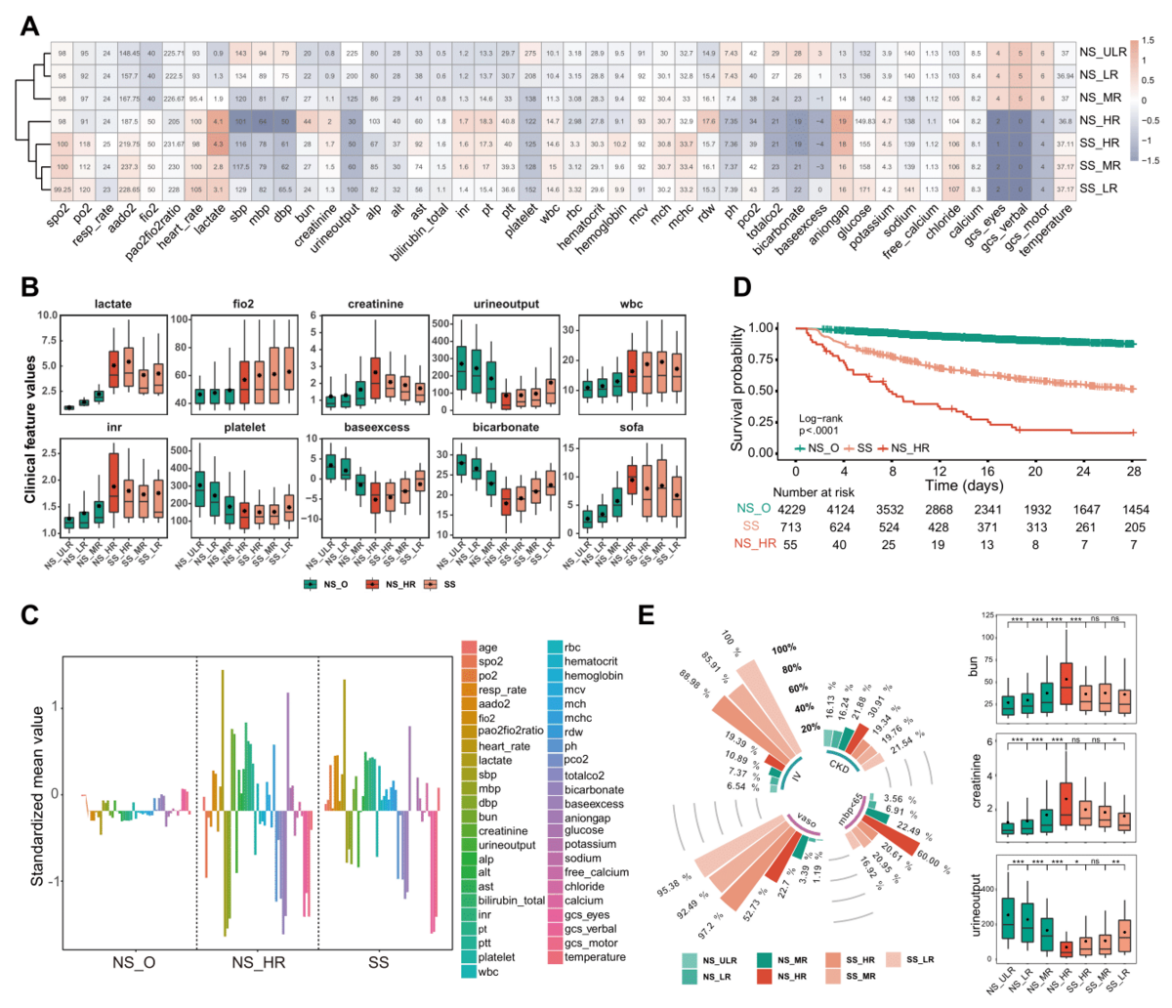
Characteristics of septic nonshock high risk (NS_HR) patients. (A) Clustering heatmap showing the similarity among risk groups. Standardized values were used for clustering (ie, each feature is centered at the sample mean and scaled by its SD), and “Euclidean” and “average” were used for clustering distance and clustering method, respectively. The values in the box are the median raw values of the features. (B) Boxplots showing the distribution of clinical features across risk groups. Red indicates NS_HR patients, orange indicates septic shock (SS) patients, and green indicates septic nonshock (NS) patients. (C) The degree of variation in the features of the risk groups. The y-axis shows the standardized mean for each clinical feature. (D) Overall survival for SS, NS_HR, and NS_O (other risk groups of NS). (E) Bar plot of the polar coordinate showing the proportion of clinical events in each risk group of patients. The boxplot shows the values of kidney-related features during the look-back interval. We transformed the infusion volume (IV), used SS_LR’s IV as a reference (3815.25 mL), and expressed the IV in other risk groups as a percentage of that of SS_LR patients. CKD: the rate of patients with chronic kidney disease; HR: high risk; IV: the mean infusion volume of patients from the preonset interval to the postonset interval; LR: low risk; MBP <65: the rate of patients with mean blood pressure (MBP) <65 mmHg from the preonset interval to the postonset interval; MR: medium risk; ns: not significant (*P*>.05); ULR: ultralow risk; vaso: the rate of patients treated with vasopressors from the preonset interval to the postonset interval. *P≤.05, **P≤.01, ***P≤.001.

Among the HR patients identified by the SORP, 11.5% (56/489) did not meet the SS definition according to the Sepsis-3 criteria [1] and were labeled NS_HR. To further confirm the disease status of these patients, the distribution of features in the postonset interval for NS_HR patients was compared to that for SS and other NS (NS_O) patients. In clustering analysis and heatmap visualization, NS_HR patients clustered with the SS groups but not with the other 3 NS groups (Figure 4A). This result indicates that the NS_HR group was more similar to SS patients in terms of clinical features, such as lactate, the SOFA score, and features related to renal function (creatinine and urine output), coagulation (prothrombin time, international normalized ratio, and platelet count), and acid-base balance (base excess, bicarbonate, total CO_2_, and anion gap) (Figure 4B). Compared to NS_O patients, both NS_HR and SS patients displayed a consistently higher degree of variation in clinical features (Figure 4C). The distribution and variation of features demonstrated that both HR_NS and SS patients had similar levels of disorder compared with NS_O patients. Moreover, both NS_HR and SS patients had significantly shorter overall survival than NS_O patients (Figure 4D). In total, NS_HR patients were highly similar to SS patients and distinct from NS_O patients. In addition, we queried the patients’ ICD codes, and 35% (19/55) of NS_HR patients were labeled with “septic shock,” which implied that a proportion of NS_HR patients might meet the previous criteria for SS.

Notably, the overall survival of NS_HR patients was significantly worse than that of SS patients (Figure 4D), and we explored the potential factors contributing to this survival difference. SS-associated treatments, such as vasopressors and fluid resuscitation, were evaluated. Compared to the SS group, the NS_HR group had a significantly higher incidence of mean blood pressure (MBP) <65 mmHg and a significantly lower rate of vasopressor use and infusion volume (all *P* values <**.**001; Figure 4E; Multimedia Appendix 8). Furthermore, NS_HR patients showed the highest percentage of CKD (*P*=**.**006) and the most abnormal kidney-related features (Figure 4E; Multimedia Appendix 8). According to this finding, one of the reasons for high mortality in NS_HR patients might be inadequate treatments, including both vasopressor use and infusion. One of the reasons NS_HR patients did not receive adequate fluid resuscitation might be severe kidney injury, as physicians may reduce fluid infusion in these patients to protect them from further severe kidney damage.

### Application of the SORP in Real-Time Risk Monitoring for SS

**Figure 5:**
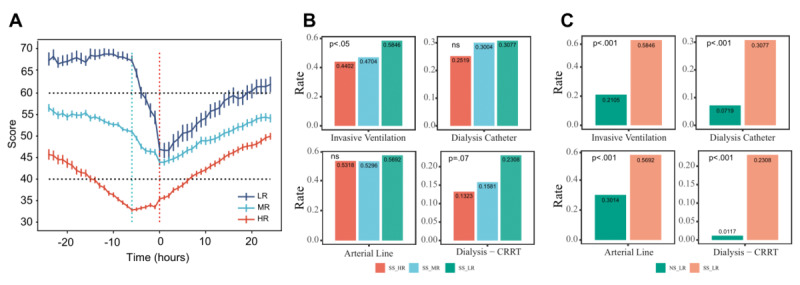
Characteristics of septic shock low risk (SS_LR) patients. (A) Mean value line and unbiased standard error bar for septic shock (SS) patients. Risk groups derived by the Septic Shock Risk Predictor (SORP) 6 hours before SS, and the change in risk over time for each group. The cyan dashed line represents 6 hours before the onset of SS, and the red dashed line represents the onset time of SS. (B) The rate of invasive procedures of each risk group in SS patients. *P* values are derived from SS_LR patients compared to other SS patients (details in Multimedia Appendix 10). (C) The rate of invasive procedures of each risk group in LR patients identified by the SORP. Here, events are measured from 12 hours before the prediction to the time of the prediction. *P* values are derived from SS_LR patients compared to other septic nonshock low risk (NS_LR) patients (details in Multimedia Appendix 11). CRRT: continuous renal replacement therapy; HR: high risk; LR: low risk; MR: medium risk; ns: not significant.

The SORP showed great performance in sepsis stratification, and we further evaluated its feasibility as a real-time monitoring risk score for patients with sepsis. The hourly risk score for SS onset was calculated by the SORP, and the patient’s risk scores and trends throughout the ICU process were fully displayed. Two patients, one with NS and another with SS, are shown in Multimedia Appendix 9. The NS patient had a progressively lower risk (increasing score) nearing discharge. In comparison, the SS patient had an increasingly higher risk until SS onset, with entry into the HR zone 6 hours before onset, and a gradual reduction in risk after onset, which might also reflect the role of treatment. Furthermore, based on continuous real-time scoring, we found that 50.0% (383/766) and 74.9% (574/766) of SS patients with risk scoring entered the HR zone 13 hours and 6 hours before onset (IQR 6-26), respectively. In addition, hourly risk scores were shown for each risk group during the 48-hour period before and after SS onset (Figure 5A). The risk gradually increased (risk score gradually decreased) as septic patients progressed to shock and decreased with treatment intervention (after SS onset), demonstrating that the SORP can monitor the patient’s risk for SS in real-time (Figure 5A).

In contrast to the gradual increase in risk in the other 2 groups, SS_LR patients maintained a relatively stable risk during and before the look-back interval but demonstrated an extremely rapid increase in risk (risk scores decreased) in the preshock interval (Figure 5A). Further analysis demonstrated that SS_LR patients had a higher rate of invasive procedures than other SS patients (Figure 5B; Multimedia Appendix 10), which was significantly higher than that of other LR patients (Figure 5C; Multimedia Appendix 11). Therefore, we speculated that this might be one of the reasons for the rapid progression of SS_LR patients: invasive procedures can introduce additional microorganisms into the body, which can exacerbate the infectious response and lead to rapid deterioration. Analysis showed that 52% (35/67) of SS_LR patients were recognized by the SORP as their risk group increased from LR to MR 3 hours before SS onset. Therefore, we recommend that LR patients exposed to invasive procedures should be assessed more frequently for the timely recognition of SS.

### Reliability and Robustness of the SORP

**Figure 6:**
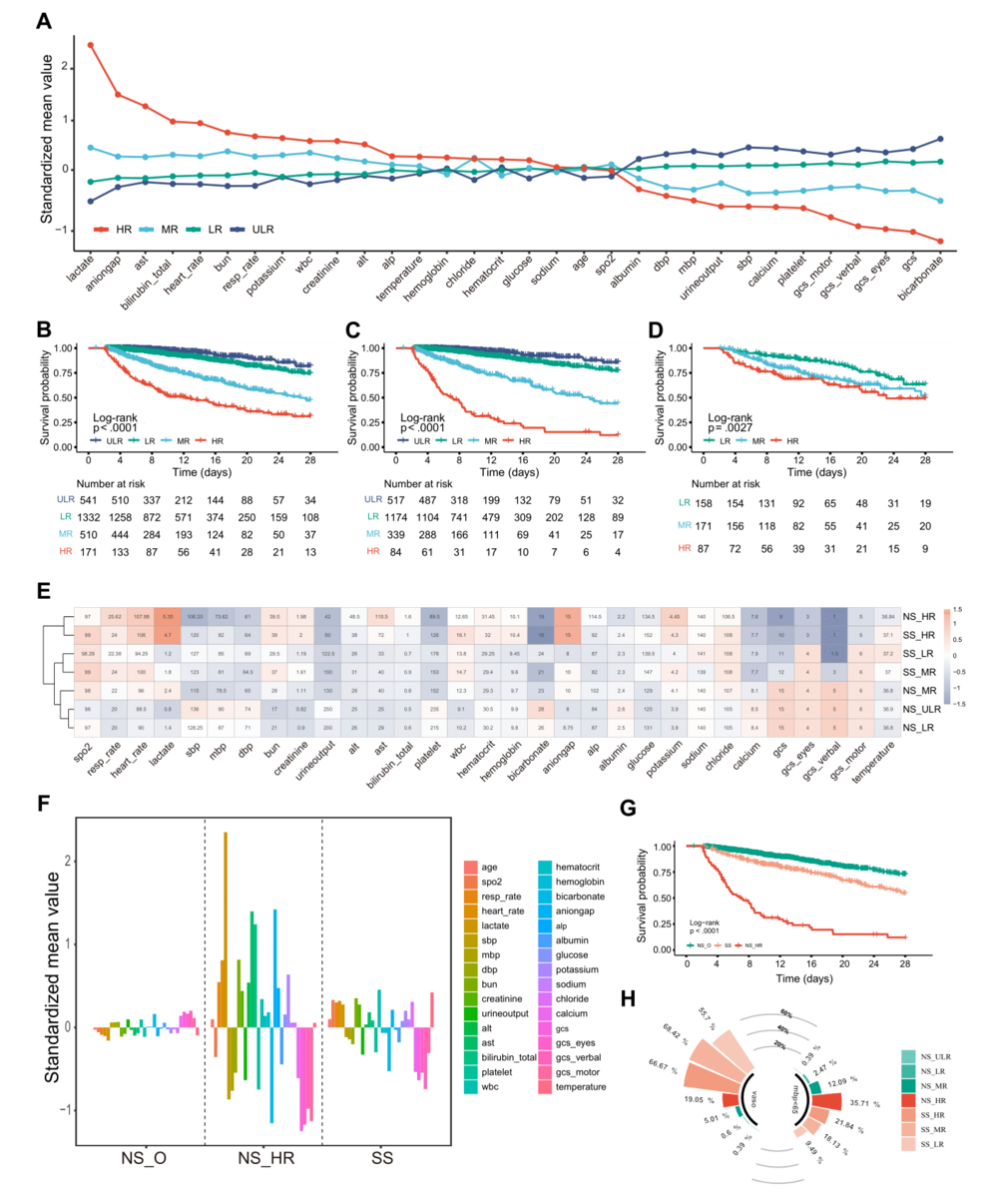
Reproducibility of findings in external independent eICU Collaborative Research Database (eICU) data. (A) The clinical features are standardized with all means scaled to 0 and SDs to 1. A value of 1 for the standardized mean value (y-axis) signifies that the mean value for the risk group was 1 SD higher than the mean value for the 4 risk groups shown in the graph. (B-D) Overall survival for all patients, septic nonshock (NS) patients, and septic shock (SS) patients. (E) Clustering heatmap showing the similarity among risk groups. Standardized values were used for clustering (ie, each feature is centered at the sample mean and scaled by its SD), and “Euclidean” and “average” were used for clustering distance and clustering method, respectively. Red indicates higher levels, and blue indicates lower levels. (F) Characteristics of risk groups. The y-axis shows the standardized mean for each clinical feature. (G) Overall survival for SS, NS_HR, and NS_O (other risk groups of NS). (H) The bar plot of the polar coordinate shows the proportion of clinical events in each risk group of patients. The starting and ending times of patient infusions are not provided in the eICU, and thus, it was not possible to estimate the infusion volume of patients within a specific interval. HR: high risk; LR: low risk; MBP <65: the rate of patients with mean blood pressure (MBP) <65 mmHg from the preonset interval to the postonset interval; MR: medium risk; ULR: ultralow risk; vaso: the rate of patients treated with vasopressors from the preonset interval to the postonset interval.

To further verify these findings, we additionally used multicenter eICU data for validation. Consistently, in the look-back interval, eICU patients were grouped into 4 risk groups. Although the proportion of SS patients was reduced in the HR group in the postonset interval (Multimedia Appendix 12), which might be due to different definitions, the distribution of SS was consistent and stable in both the eICU dataset and the MIMIC-IV dataset (PSI <0**.**05; Multimedia Appendix 13). Similar to the MIMIC-IV, the patients’ features became increasingly abnormal as risk increased, especially features related to the SS definition (eg, lactate and blood pressure), kidneys, acid-base balance, and coagulation (Figure 6A; Multimedia Appendix 14). For overall survival, the 4 risk groups also showed significant differences, even in NS patients and SS patients (Figure 6B-D). SS_LR patients, similar to MIMIC-IV patients, underwent significantly more invasive procedures than the other LR patients (Multimedia Appendix 15), but there was no significant difference from the other SS patients (Multimedia Appendix 16). NS_HR patients, similar to MIMIC-IV patients, significantly differed from NS_O patients in clinical features and survival, being more similar to SS patients but with a more severe status (Figure 6E-G). Regarding treatment, as the eICU did not provide start and end times for infusion, we could not obtain accurate infusion volumes for eICU patients within a certain interval, so we focused only on vasopressor use. Compared to SS patients, NS_HR patients showed significant hypotension and less vasopressor use, which were similar to the results of the MIMIC-IV (Figure 6H; Multimedia Appendix 17). Although this analysis was based on a different definition of SS, similar results were still observed, further confirming the robustness of the SORP and the reasonability of the risk stratification system.

In summary, the SORP has a reliable and robust ability to not only provide early forewarning but also stratify patients with potentially different risks of SS. For convenience, we provide an online service of the SORP [28]. The “Help” section in the navigation bar of the website contains detailed instructions on how to use the online tool.

## Discussion

### Principal Findings

Timely forewarning and early intervention are especially important for patients who are developing SS, and clinically applicable forewarning tools for SS are still lacking. In this study, we constructed the SORP, a simplified model, that not only provides real-time risk stratification for patients but can also easily trace the SS risk of patients during the entire ICU process and forewarn SS onset with a median of 13 hours in advance. The SORP can precisely assess the risk of SS 6 hours in advance by using only ABG and VS features, with an AUC of 0**.**9458 in the test set. Compared to the findings in previous studies, the SORP remarkably reduces both time and economic costs by using easily available ABG and VS features that can be accessed within minutes and can achieve competitive predictive performance (Multimedia Appendix 18). In contrast, other studies require additional tests, such as biochemical tests, SOFA score calculations, and specific urine output measurements, which extend data collection by several hours. While some studies reduce the number of features through feature engineering methods, these features may still be distributed across test batches, which may not effectively shorten the overall time and costs needed for assessments. Moreover, in addition to being available through the web service [28], the patient’s risk score can be simply and quickly calculated manually using a scorecard (Multimedia Appendix 2). The ease of data collection and the simplicity of use will greatly enhance the usefulness of the SORP in clinical applications. Furthermore, we focused more on the clinical characteristics of HR patients, especially septic NS patients, and the assessment of newly discovered NS_HR patients, overlooked by the Sepsis-3 definition, may provide further insights into the pathophysiological processes of SS onset and help to complement its diagnosis and precise management.

In this study, 4 risk groups were identified by the SORP. For these risk groups, we observed distinct clinical features and a significantly stratified risk for SS onset rates and survival status. For SS patients in different risk groups, the time from sepsis onset to SS decreased as their risk increased. Significant survival differences were observed in NS patients across different risk groups, but these same differences were not observed in SS patients (Figure 3E). This may be because of the active guideline-directed resuscitation treatment of SS patients. These findings confirm the effectiveness of the current SS resuscitation treatment guidelines and further affirm the accuracy of risk stratification and the reliability of the SORP.

Interestingly, despite the high agreement between SS patients diagnosed by the Sepsis-3 definition and those identified by the SORP, we still identified 2 relatively distinct groups. One group was SS_LR, where patients were defined as having SS by Sepsis-3 but were identified as LR by the SORP. The overall survival of these patients was not significantly different from that of other SS groups, but rapid progression and an extremely increased risk were demonstrated within 6 hours before SS onset (Figure 5A). In addition, 52% (35/67) of SS_LR patients showed an increase from LR to MR 3 hours before SS onset. Thus, although these patients could not be identified 6 hours in advance, their risk changes were captured in real-time, which highlights the high sensitivity of the SORP. Further analysis showed that SS_LR patients underwent more invasive procedures than other SS patients and much more than other patients in the LR group, such as “invasive ventilation,” “dialysis catheter,” “arterial line,” and “dialysis-CRRT” (Figure 5B and 5C). Thus, we speculate that invasive procedures might be one of the causes of the rapidly exacerbating patient status. We recommend that LR patients undergoing invasive procedures should be evaluated more frequently to recognize rapid exacerbation in a timely manner.

Another notable group was NS_HR, where patients were not defined as having SS by Sepsis-3 but were identified as HR by the SORP. This prompted us to further analyze this particular patient group, and the results showed that these patients had the following characteristics: (1) NS_HR patients were quite similar to SS patients in terms of clinical phenotypes (Figure 4A-C); (2) NS_HR patients had the worst overall survival, which was significantly poorer than even that of SS_HR patients (Figure 3C and 3D; Figure 4D); (3) NS_HR patients did not receive sufficient fluid resuscitation or timely vasopressor administration (Figure 4E), which might be a key factor that resulted in their noncompliance with the SS diagnosis and their higher mortality; and (4) NS_HR patients had more significant abnormalities in renal-related features (Figure 4E), which reflect higher incidences of renal malfunction, such as CKD (Figure 4E). The literature has shown that excessive fluid resuscitation in patients with severe renal disease exacerbates renal injury and leads to poorer outcomes [33,34]. Therefore, cautious and conservative strategies might be adopted by physicians, resulting in a much higher likelihood of insufficient fluid resuscitation in these patients. Hence, precise fluid resuscitation management is crucial in SS patients with renal dysfunction. In total, NS_HR patients share a similar pathophysiologic process with SS patients but experience a more aggressive clinical outcome due to insufficient treatment.

Compared to previous versions, the Sepsis-3 shock definition is more focused on tissue-organ hypoperfusion and metabolic dysfunction and the corresponding therapeutic response, which largely ensures the authenticity and reliability of SS diagnosis, showing a significant increase in specificity but at the cost of sensitivity [35]. Studies have shown that a proportion of patients who met the previous version of the SS criteria, but not the Sepsis-3 shock criteria, had significant organ failure and high mortality [36], with a value of up to 31**.**6% [37]. In this study, we identified a group of patients (NS_HR) with poor survival and a similar clinical phenotype to SS. In addition to low vasopressor use, the NS_HR group had inadequate fluid resuscitation due to severe renal disease, thus failing the Sepsis-3 shock definition. These patients are missed by the current SS definition, but they are at HR and are worthy of attention. Therefore, for more precise diagnosis and management of SS patients, “adequate fluid resuscitation” in the SS definition may have different criteria in different populations. The need for fluid personalization has emerged as an admirable goal, especially in SS [38].

### Limitations

In this study, the designed SS risk early forewarning and stratification model, SORP, can easily and quickly provide ICU physicians with the patient’s risk status and its changing trend, which can provide valuable support for the early identification of patient deterioration. However, it should be noted that there are limitations in this study. The current data sources are relatively limited in terms of geographic regions, which to some extent hinders the study of differences in different regions. Future studies should collect data from different regions to test the accuracy of the SORP for early forewarning, analyze the impact of regional differences on risk factors for SS, and establish a more accurate early forewarning system. Moreover, it is crucial to regularly monitor and assess the early forewarning capability and stability of the system and to make necessary adjustments as additional data becomes available. In addition, in the eICU dataset, we were unable to define SS by the Sepsis-3 criteria due to data limitations, which resulted in the incomparability of performance metrics such as AUC. However, based on a different SS definition, we were still able to observe results similar to those with the MIMIC-IV data, which further confirms the robustness of the SORP and the rationality of the risk stratification system.

### Summary and Outlook

Early forewarning and diagnosis of SS are essential for reducing mortality. To support this, we developed the SORP early forewarning model, which offers several key benefits: (1) easy and rapid feature collection within minutes; (2) ease of use via online tools or a scorecard (Multimedia Appendix 2); (3) excellent predictive performance (Figure 2B); (4) continuous status monitoring for risk deterioration or improvement; and (5) identification of HR patients, including those with similar symptoms who may not fully meet diagnostic criteria. In clinical practice, the SORP can be easily integrated into sepsis management as an auxiliary monitoring tool (Multimedia Appendix 19). For MR patients whose SORP score is still decreasing, it is recommended to increase the frequency of blood gas testing for timely monitoring. For those identified as HR, the SS determination process can be initiated ahead of time, facilitating the early administration of appropriate treatments, such as fluid resuscitation.

Future research should further explore the applicability of the SORP model in diverse clinical settings, including validation with additional datasets. We have also planned a prospective trial to evaluate its impact on prognosis and survival, particularly in NS_HR patients. These efforts aim to advance early SS identification and management, ultimately improving patient outcomes.

## Appendix

Multimedia Appendix 1 Checklist according to the TRIPOD (Transparent Reporting of a Multivariable Prediction Model for Individual Prognosis or Diagnosis) guidelines.

Multimedia Appendix 2 Septic shock risk scorecard for the Septic Shock Risk Predictor.

Multimedia Appendix 3 Performance of deep learning and machine learning models.

Multimedia Appendix 4 Distribution stability of septic shock in Medical Information Mart for Intensive Care-IV (MIMIC-IV) for different risk groups.

Multimedia Appendix 5 Medical Information Mart for Intensive Care-IV (MIMIC-IV) data for the rate of patients with septic shock in each risk group.

Multimedia Appendix 6 The onset rate of septic shock in each risk score according to the fitted curve.

Multimedia Appendix 7 Boxplots showing change trends for each clinical feature by risk groups in Medical Information Mart for Intensive Care-IV (MIMIC-IV) data.

Multimedia Appendix 8 Medical Information Mart for Intensive Care-IV (MIMIC-IV) data for the clinical event distribution and significance of risk groups.

Multimedia Appendix 9 The changing trends in risk across the whole intensive care unit (ICU) process.

Multimedia Appendix 10 Medical Information Mart for Intensive Care-IV (MIMIC-IV) data for the invasive operation distribution and the significance of septic shock risk groups.

Multimedia Appendix 11 Medical Information Mart for Intensive Care-IV (MIMIC-IV) data for the invasive operation distribution and the significance of low-risk groups.

Multimedia Appendix 12 eICU Collaborative Research Database (eICU) data for the rate of patients with septic shock in each risk group.

Multimedia Appendix 13 Distribution stability of septic shock between the eICU Collaborative Research Database (eICU) and Medical Information Mart for Intensive Care-IV (MIMIC-IV) datasets.

Multimedia Appendix 14 Boxplots showing change trends for each clinical feature by risk groups in the eICU Collaborative Research Database (eICU) data.

Multimedia Appendix 15 eICU Collaborative Research Database (eICU) data for the invasive operation distribution and the significance of low-risk groups.

Multimedia Appendix 16 eICU Collaborative Research Database (eICU) data for the invasive operation distribution and the significance of septic shock risk groups.

Multimedia Appendix 17 eICU Collaborative Research Database (eICU) data for the clinical event distribution and the significance of risk groups.

Multimedia Appendix 18 Current studies for predicting the onset of septic shock.

Multimedia Appendix 19 Schematic diagram for auxiliary monitoring of the Septic Shock Risk Predictor (SORP) in clinical practice.

## Abbreviations

ABG: arterial blood gas
AUC: area under the curve
BIDMC: Beth Israel Deaconess Medical Center
CKD: chronic kidney disease
CRRT: dialysis-continuous renal replacement therapy
eICU: eICU Collaborative Research Database
HR: high risk
ICD: International Classification of Diseases
ICU: intensive care unit
IV: information value
KS: Kolmogorov-Smirnov
LogR: logistic regression
LR: low risk
MBP: mean blood pressure
MIMIC-IV: Medical Information Mart for Intensive Care-IV
MR: medium risk
NS: nonshock
NS_O: other nonshock patients, excluding NS_HR
PSI: Population Stability Index
SHAP: Shapley Additive Explanations
SOFA: Sequential Organ Failure Assessment
SORP: Septic Shock Risk Predictor
SS: septic shock
ULR: ultralow risk
VS: vital sign
WOE: weight of evidence

## Data Availability

The data generated and analyzed during this study are available from the corresponding author upon request. The electronic health record data used in this study are openly available and can be downloaded at the MIMIC-IV [30] and eICU [39].
