# Supplementary material for: An Easy and Quick Risk-Stratified Early Forewarning Model for Septic Shock in the Intensive Care Unit: Development, Validation, and Interpretation Study
Source: J Med Internet Res. 2025 Feb 6;27:e58779. doi: 10.2196/58779 (PMC11843061; doi:10.2196/58779)
Supplement: Multimedia Appendix 1 [file jmir_v27i1e58779_app1.docx]

# Multimedia Appendix 1. Checklist according to the TRIPOD (Transparent Reporting of a Multivariable Prediction Model for Individual Prognosis or Diagnosis) guidelines.

| **Section/Topic** | **Checklist item** | **Repoted on page / Describtion** |
| --- | --- | --- |
| **Methods** | |  |
| *Data* | Describe the sources of data separately for the development and evaluation datasets (e.g., randomised trial, cohort, routine care or registry data), the rationale for using these data, and representativeness of the data | Data were collected from the open-source datasets MIMIC-IV and eICU. For more information, please refer to the "Datasets and Participants" section in "Materials and Methods", or the corresponding official websites: MIMIC-IV (https://mimic. mit.edu/) and eICU (https://eicu-crd.mit.edu/). |
|  | Specify the dates of the collected participant data, including start and end of participant accrual; and, if applicable, end of follow-up |  |
| *Participants* | Specify key elements of the study setting (e.g., primary care, secondary care, general population) including the number and location of centres |  |
|  | Describe the eligibility criteria for study participants |  |
|  | Give details of any treatments received, and how they were handled during model development or evaluation, if relevant |  |
| *Data preparation* | Describe any data pre-processing and quality checking, including whether this was similar across relevant sociodemographic groups | "Data processing" section of Materials and Methods |
| *Outcome* | Clearly define the outcome that is being predicted and the time horizon, including how and when assessed, the rationale for choosing this outcome, and whether the method of outcome assessment is consistent across sociodemographic groups | The predicted positive sample was septic shock, which was defined as shown in the ‘Definitions of sepsis and SS’ section. For the different risk groups given by the model, estimation and comparison of the survival curves among risk groups were performed using Kaplan‒Meier survival analysis and log-rank tests, respectively. Of these, all-cause death was the outcome, and specifically, for SS patients, we further evaluated the differences among risk groups using the time from sepsis to septic shock. |
|  | If outcome assessment requires subjective interpretation, describe the qualifications and demographic characteristics of the outcome assessors | Not applicable |
|  | Report any actions to blind assessment of the outcome to be predicted | Not applicable |
| *Predictors* | Describe the choice of initial predictors (e.g., literature, previous models, all available predictors) and any pre-selection of predictors before model building | The features selected for modelling are described in the ‘Development of the septic shock risk predictor (SORP)’ section of the Results. |
|  | Clearly define all predictors, including how and when they were measured (and any actions to blind assessment of predictors for the outcome and other predictors) | The use of the model is mentioned in the "Summary and Outlook" section of the discussion. And, Fig. S6 presents a schematic diagram for auxiliary monitoring of SORP in clinical practice, mentioning the time of clinical features measurement. |
|  | If predictor measurement requires subjective interpretation, describe the qualifications and demographic characteristics of the predictor assessors | Not applicable |
| *Sample size* | Explain how the study size was arrived at (separately for development and evaluation), and justify that the study size was sufficient to answer the research question. Include details of any sample size calculation | Not applicable |
| *Missing data* | Describe how missing data were handled. Provide reasons for omitting any data | "Data processing" section of Materials and Methods |
| *Analytical methods* | Describe how the data were used (e.g., for development and evaluation of model performance) in the analysis, including whether the data were partitioned, considering any sample size requirements | The second paragraphs of "Datasets and participants" section in Materials and Methods |
|  | Depending on the type of model, describe how predictors were handled in the analyses (functional form, rescaling, transformation, or any standardisation). | "Risk model" section of Materials and Methods |
|  | Specify the type of model, rationale2, all model-building steps, including any hyperparameter tuning, and method for internal validation | "Risk model" section of Materials and Methods |
|  | Describe if and how any heterogeneity in estimates of model parameter values and model performance was handled and quantified across clusters (e.g., hospitals, countries). See TRIPOD-Cluster for additional considerations3 | Not applicable |
|  | Specify all measures and plots used (and their rationale) to evaluate model performance (e.g., discrimination, calibration, clinical utility) and, if relevant, to compare multiple models | "Statistical Methods" section of Materials and Methods |
|  | Describe any model updating (e.g., recalibration) arising from the model evaluation, either overall or for particular sociodemographic groups or settings | Not applicable |
|  | For model evaluation, describe how the model predictions were calculated (e.g., formula, code, object, application programming interface) | Model construction is illustrated in "Risk model" section of Materials and Methods. And the second paragraph in ‘Statistical Methods’ mentions how the risk scores output from the model are transformed into risk intervals for risk stratification of patients. |
| *Class imbalance* | If class imbalance methods were used, state why and how this was done, and any subsequent methods to recalibrate the model or the model predictions | Not applicable |
| *Fairness* | Describe any approaches that were used to address model fairness and their rationale | Not applicable |
| *Model output* | Specify the output of the prediction model (e.g., probabilities, classification). Provide details and rationale for any classification and how the thresholds were identified | Model construction is illustrated in "Risk model" section of Materials and Methods. And the second paragraph in ‘Statistical Methods’ mentions how the risk scores output from the model are transformed into risk intervals for risk stratification of patients. More explanation of the model output is detailed in the ‘Development of septic shock risk predictors (SORP)’ section of ‘Results’. |
| *Training versus evaluation* | Identify any differences between the development and evaluation data in healthcare setting, eligibility criteria, outcome, and predictors | "Datasets and participants" section of Materials and Methods |
| *Ethical approval* | Name the institutional research board or ethics committee that approved the study and describe the participant-informed consent or the ethics committee waiver of informed consent | "Ethical Considerations" section of Materials and Methods |
