# Supplementary material for: An Easy and Quick Risk-Stratified Early Forewarning Model for Septic Shock in the Intensive Care Unit: Development, Validation, and Interpretation Study
Source: J Med Internet Res. 2025 Feb 6;27:e58779. doi: 10.2196/58779 (PMC11843061; doi:10.2196/58779)
Supplement: Multimedia Appendix 2 [file jmir_v27i1e58779_app2.docx]

# Multimedia Appendix 2. Septic shock risk scorecard for the Septic Shock Risk Predictor.

| feature  Base Score: 57 | bin | score | feature | bin | score | feature | bin | score |
| --- | --- | --- | --- | --- | --- | --- | --- | --- |
| heart_rate | [-inf,108.0] | 0 | mbp | [-inf,64.0] | -1 | glucose | [-inf,170.0] | 1 |
|  | [108.0,inf] | -1 |  | [64.0,74.0] | 0 |  | [170.0,185.0] | -1 |
| resp_rate | [-inf,10.0] | 1 |  | [74.0,84.0] | 1 |  | [185.0,inf] | -2 |
|  | [10.0,20.0] | 0 |  | [84.0,inf] | 2 | hematocrit | [-inf,27.0] | 0 |
|  | [20.0,inf] | -1 | sbp | [-inf,90.0] | -5 |  | [27.0,31.0] | 1 |
| temperature | [-inf,36.3] | -3 |  | [90.0,105.0] | -1 |  | [31.0,37.0] | 0 |
|  | [36.3,36.7] | -1 |  | [105.0,115.0] | 2 |  | [37.0,inf] | -1 |
|  | [36.7,37.4] | 1 |  | [115.0,inf] | 6 | sodium | [-inf,134.0] | -1 |
|  | [37.4,38.2] | 0 | lactate | [-inf,1.0] | 19 |  | [134.0,inf] | 0 |
|  | [38.2,inf] | -4 |  | [1.0,1.2] | 12 | potassium | [-inf,3.4] | 0 |
| spo2 | [-inf,91.0] | -1 |  | [1.2,2.3] | 4 |  | [3.4,4.2] | 2 |
|  | [91.0,96.0] | 2 |  | [2.3,3.9] | -8 |  | [4.2,4.9] | 0 |
|  | [96.0,99.0] | 0 |  | [3.9,inf] | -19 |  | [4.9,inf] | -4 |
|  | [99.0,100.0] | -3 | bicarbonate | [-inf,18.0] | -9 | hemoglobin | [-inf,9.0] | 0 |
|  | [100.0,inf] | -5 |  | [18.0,23.0] | -3 |  | [9.0,10.0] | 1 |
| dbp | [-inf,38.0] | -1 |  | [23.0,26.0] | 1 |  | [10.0,13.0] | 0 |
|  | [38.0,64.0] | 0 |  | [26.0,28.0] | 3 |  | [13.0,inf] | -2 |
|  | [64.0,inf] | 1 |  | [28.0,inf] | 6 | calcium | [-inf,8.0] | -1 |
|  |  |  |  |  |  |  | [8.0,inf] | 0 |
