# Supplementary material for: An Easy and Quick Risk-Stratified Early Forewarning Model for Septic Shock in the Intensive Care Unit: Development, Validation, and Interpretation Study
Source: J Med Internet Res. 2025 Feb 6;27:e58779. doi: 10.2196/58779 (PMC11843061; doi:10.2196/58779)
Supplement: Multimedia Appendix 3 [file jmir_v27i1e58779_app3.docx]

# Multimedia Appendix 3. Performance of deep learning and machine learning models.

| **models** | **Validation set** | | | | | **Test set** | | | | |
| --- | --- | --- | --- | --- | --- | --- | --- | --- | --- | --- |
|  | **Sensitivity** | **Specificity** | **Accuracy** | **F1** | **AUC** | **Sensitivity** | **Specificity** | **Accuracy** | **F1** | **AUC** |
| **Deep learning models** | | | | | | | | | | |
| **TCN** | 92.02% | 89.39% | 89.79% | 73.35% | 93.97% | 92.67% | 89.34% | 89.80% | 71.83% | 94.23% |
| **BiGRU** | 87.12% | 92.82% | 91.95% | 76.76% | 94.16% | 84.67% | 91.40% | 90.46% | 71.35% | 94.46% |
| **GRU** | 90.80% | 90.94% | 90.92% | 75.32% | 94.63% | 87.33% | 91.19% | 90.65% | 72.38% | 95.45% |
| **LSTM** | 91.41% | 91.27% | 91.29% | 76.21% | 94.73% | 86.67% | 89.12% | 88.77% | 68.42% | 95.00% |
| **BiLSTM** | 88.96% | 92.93% | 92.32% | 77.96% | 95.19% | 88.00% | 91.29% | 90.83% | 72.93% | 95.98% |
| **Machine learning models** | | | | | | | | | | |
| **CatBoost_BAG_L1** | 91.72% | 93.30% | 93.07% | 78.93% | **96.40%** | 89.02% | 94.54% | 93.66% | 81.79% | **96.51%** |
| **RandomForestEntr_BAG_L2** | 82.76% | 95.34% | 93.56% | 78.43% | 96.38% | 76.22% | 96.86% | 93.56% | 79.11% | 96.59% |
| **LightGBMXT_BAG_L1** | 83.45% | 94.66% | 93.07% | 77.32% | 96.33% | 82.32% | 96.05% | 93.85% | 81.08% | 96.48% |
| **LightGBMXT_BAG_L2** | 79.31% | 95.91% | 93.56% | 77.70% | 96.27% | 75.00% | 97.21% | 93.66% | 79.10% | 96.61% |
| **WeightedEnsemble_L2** | 88.97% | 94.09% | 93.37% | 79.14% | 96.26% | 82.93% | 95.47% | 93.46% | 80.24% | 96.48% |
| **LightGBM_BAG_L1** | 82.07% | 95.80% | 93.85% | 79.07% | 96.19% | 76.83% | 96.86% | 93.66% | 79.50% | 96.45% |
| **XGBoost_BAG_L1** | 90.34% | 94.77% | 94.15% | 81.37% | 96.18% | 79.88% | 95.35% | 92.88% | 78.21% | 96.44% |
| **CatBoost_BAG_L2** | 83.45% | 95.11% | 93.46% | 78.32% | 96.17% | 78.66% | 96.86% | 93.95% | 80.63% | 96.55% |
| **WeightedEnsemble_L3** | 86.21% | 95.00% | 93.76% | 79.62% | 96.15% | 80.49% | 96.17% | 93.66% | 80.24% | 96.53% |
| **XGBoost_BAG_L2** | 86.90% | 94.77% | 93.66% | 79.50% | 96.12% | 84.76% | 95.70% | 93.95% | 81.76% | 96.38% |
| **RandomForestGini_BAG_L2** | 86.90% | 94.89% | 93.76% | 79.75% | 96.11% | 80.49% | 96.05% | 93.56% | 80.00% | 96.39% |
| **LightGBMLarge_BAG_L2** | 88.28% | 94.32% | 93.46% | 79.26% | 96.11% | 85.37% | 95.47% | 93.85% | 81.63% | 96.27% |
| **LightGBM_BAG_L2** | 88.97% | 93.86% | 93.17% | 78.66% | 96.07% | 87.20% | 94.66% | 93.46% | 81.02% | 96.63% |
| **RandomForestEntr_BAG_L1** | 64.14% | 97.95% | 93.17% | 72.66% | 96.04% | 62.20% | 98.03% | 92.29% | 72.08% | 96.21% |
| **NeuralNetFastAI_BAG_L2** | 86.21% | 93.75% | 92.68% | 76.92% | 95.99% | 84.76% | 94.66% | 93.07% | 79.66% | 96.38% |
| **RandomForestGini_BAG_L1** | 65.52% | 98.07% | 93.46% | 73.93% | 95.97% | 64.02% | 98.14% | 92.68% | 73.68% | 96.52% |
| **ExtraTreesEntr_BAG_L2** | 84.83% | 95.23% | 93.76% | 79.35% | 95.97% | 78.05% | 96.75% | 93.76% | 80.00% | 96.68% |
| **ExtraTreesGini_BAG_L2** | 84.14% | 95.34% | 93.76% | 79.22% | 95.96% | 78.66% | 96.86% | 93.95% | 80.63% | 96.81% |
| **NeuralNetFastAI_BAG_L1** | 86.90% | 93.52% | 92.59% | 76.83% | 95.78% | 84.15% | 94.31% | 92.68% | 78.63% | 96.09% |
| **NeuralNetTorch_BAG_L2** | 86.90% | 91.82% | 91.12% | 73.47% | 95.71% | 92.68% | 92.68% | 92.68% | 80.21% | 96.29% |
| **LightGBMLarge_BAG_L1** | 60.00% | 98.18% | 92.78% | 70.16% | 95.65% | 57.93% | 98.26% | 91.80% | 69.34% | 96.16% |
| **NeuralNetTorch_BAG_L1** | 84.83% | 93.64% | 92.39% | 75.93% | 95.55% | 84.15% | 93.96% | 92.39% | 77.97% | 95.90% |
| **ExtraTreesEntr_BAG_L1** | 56.55% | 97.95% | 92.10% | 66.94% | 95.25% | 55.49% | 98.61% | 91.71% | 68.16% | 95.93% |
| **ExtraTreesGini_BAG_L1** | 57.93% | 97.95% | 92.29% | 68.02% | 95.03% | 55.49% | 98.37% | 91.51% | 67.66% | 96.06% |
| **KNeighborsDist_BAG_L1** | 3.45% | 100.00% | 86.34% | 6.67% | 80.80% | 3.05% | 99.77% | 84.29% | 5.85% | 83.12% |
| **KNeighborsUnif_BAG_L1** | 78.62% | 72.73% | 73.56% | 45.69% | 80.54% | 79.88% | 77.12% | 77.56% | 53.25% | 82.65% |

note: For machine learning modes: One of the newly developed automatic machine learning (AutoML) frameworks, AutoGluon [1], was applied to construct the machine learning-based models. AutoGluon settings: We set the "time limit" parameter for the entire model to 20 minutes. The evaluation metric for each model in the ensemble was set to "roc_auc". We also set the "presets" parameter to be "best_quality" to improve the ensemble models' predictive performance based on stacking and bagging in the granted training time. Here are the 32 model input features related to acid-base balance, coagulation, renal, hepatic, pulmonary, hematologic, Glasgow Coma Index, etc: gender, age, heart rate, mbp, spo2, respiration rate, sbp, dbp, temperature, urine output, gcs, gcs_eyes, gcs_verbal, gcs_motor, glucose, potassium, hematocrit, sodium, chloride, hemoglobin, creatinine, bicarbonate, bun, anion gap, platelet, wbc, calcium, bilirubin_total, alt, ast, alp, lactate.

**References**

1. Erickson N, Mueller J, Shirkov A, Zhang H, Larroy P, Li M, et al. Autogluon-tabular: Robust and accurate automl for structured data. arXiv preprint arXiv:200306505. 2020.
