# Supplementary material for: An Easy and Quick Risk-Stratified Early Forewarning Model for Septic Shock in the Intensive Care Unit: Development, Validation, and Interpretation Study
Source: J Med Internet Res. 2025 Feb 6;27:e58779. doi: 10.2196/58779 (PMC11843061; doi:10.2196/58779)
Supplement: Multimedia Appendix 4 [file jmir_v27i1e58779_app4.docx]

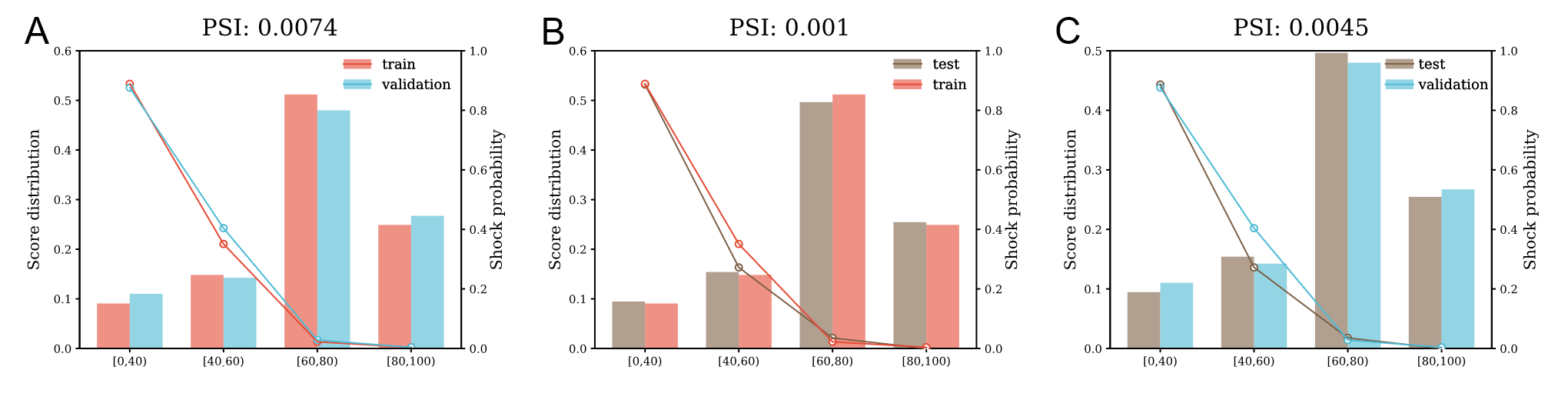


# Multimedia Appendix 4. Distribution stability of septic shock in Medical Information Mart for Intensive Care-IV (MIMIC-IV) for different risk groups. (A) for train and validation dataset. (B) for train and test dataset. (C) for validation and test dataset.
