# Supplementary material for: An Easy and Quick Risk-Stratified Early Forewarning Model for Septic Shock in the Intensive Care Unit: Development, Validation, and Interpretation Study
Source: J Med Internet Res. 2025 Feb 6;27:e58779. doi: 10.2196/58779 (PMC11843061; doi:10.2196/58779)
Supplement: Multimedia Appendix 5 [file jmir_v27i1e58779_app5.docx]

# Multimedia Appendix 5. Medical Information Mart for Intensive Care-IV (MIMIC-IV) data for the rate of patients with septic shock in each risk group.

| group | septic shock | septic non-shock | total | rate |
| --- | --- | --- | --- | --- |
| high_risk | 433 | 56 | 489 | 0.8855 |
| medium_risk | 262 | 498 | 760 | 0.3447 |
| low_risk | 67 | 2640 | 2707 | 0.0248 |
| ultra_low_risk | 4 | 1297 | 1301 | 0.0031 |
