# Supplementary material for: An Easy and Quick Risk-Stratified Early Forewarning Model for Septic Shock in the Intensive Care Unit: Development, Validation, and Interpretation Study
Source: J Med Internet Res. 2025 Feb 6;27:e58779. doi: 10.2196/58779 (PMC11843061; doi:10.2196/58779)
Supplement: Multimedia Appendix 6 [file jmir_v27i1e58779_app6.docx]

# Multimedia Appendix 6. The onset rate of septic shock in each risk score according to the fitted curve.

| Risk score | Onset rate (%) | Risk score | Onset rate (%) | Risk score | Onset rate (%) |
| --- | --- | --- | --- | --- | --- |
| ≥7 | >97 | 32 | 86 | 57 | 18 |
| 8 | 97 | 33 | 85 | 58 | 15 |
| 9 | 97 | 34 | 84 | 59 | 13 |
| 10 | 97 | 35 | 82 | 60 | 11 |
| 11 | 97 | 36 | 81 | 61 | 9 |
| 12 | 97 | 37 | 79 | 62 | 8 |
| 13 | 97 | 38 | 77 | 63 | 6 |
| 14 | 97 | 39 | 74 | 64 | 5 |
| 15 | 96 | 40 | 71 | 65 | 4 |
| 16 | 96 | 41 | 68 | 66 | 3 |
| 17 | 96 | 42 | 65 | 67 | 2 |
| 18 | 96 | 43 | 63 | 68 | 2 |
| 19 | 95 | 44 | 59 | 69 | 2 |
| 20 | 95 | 45 | 56 | 70 | 1 |
| 21 | 94 | 46 | 52 | 71 | 1 |
| 22 | 94 | 47 | 48 | 72 | 1 |
| 23 | 93 | 48 | 45 | 73 | 1 |
| 24 | 93 | 49 | 42 | 74 | 1 |
| 25 | 92 | 50 | 38 | 75 | <1 |
| 26 | 91 | 51 | 35 | 76 | <1 |
| 27 | 90 | 52 | 32 | 77 | <1 |
| 28 | 90 | 53 | 28 | 78 | <1 |
| 29 | 89 | 54 | 25 | 79 | <1 |
| 30 | 88 | 55 | 23 | ≤80 | <0.3 |
| 31 | 87 | 56 | 20 |  |  |
