# Supplementary material for: An Easy and Quick Risk-Stratified Early Forewarning Model for Septic Shock in the Intensive Care Unit: Development, Validation, and Interpretation Study
Source: J Med Internet Res. 2025 Feb 6;27:e58779. doi: 10.2196/58779 (PMC11843061; doi:10.2196/58779)
Supplement: Multimedia Appendix 7 [file jmir_v27i1e58779_app7.docx]

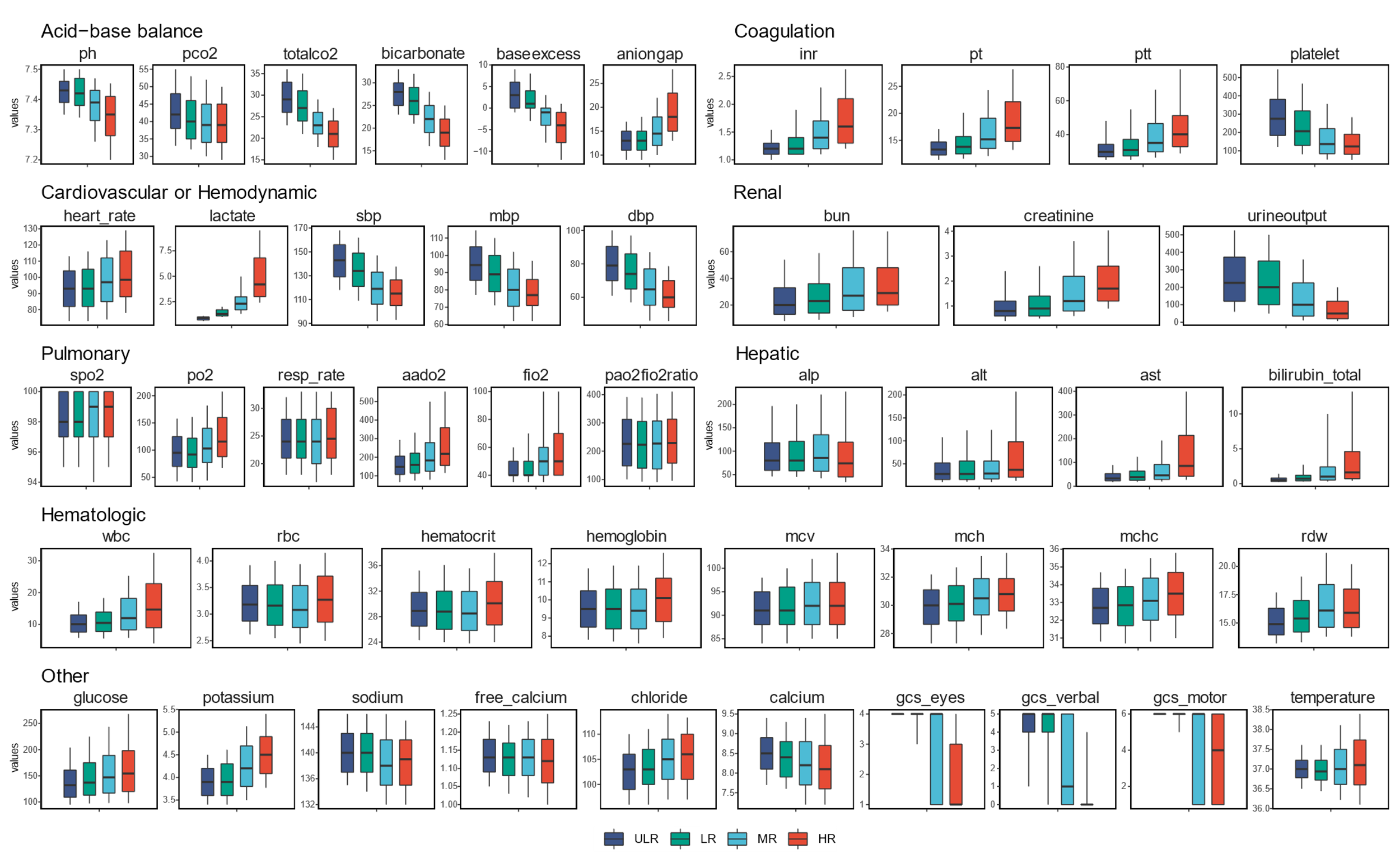


# Multimedia Appendix 7. Boxplots showing change trends for each clinical feature by risk groups in Medical Information Mart for Intensive Care-IV (MIMIC-IV) data. The y-axis shows the standardized value for each clinical feature.
