# Supplementary material for: An Easy and Quick Risk-Stratified Early Forewarning Model for Septic Shock in the Intensive Care Unit: Development, Validation, and Interpretation Study
Source: J Med Internet Res. 2025 Feb 6;27:e58779. doi: 10.2196/58779 (PMC11843061; doi:10.2196/58779)
Supplement: Multimedia Appendix 8 [file jmir_v27i1e58779_app8.docx]

# Multimedia Appendix 8. Medical Information Mart for Intensive Care-IV (MIMIC-IV) data for the clinical event distribution and significance of risk groups.

|  | [ALL] N=4992 | NS_O N=4226 | NS_HR N=55 | SS  N=711 | p.overall | p.NS_O vs NS_HR | p.NS_O vs SS | p.NS_HR vs SS |
| --- | --- | --- | --- | --- | --- | --- | --- | --- |
| vaso | 917 (18.4%) | 210 (4.97%) | 29 (52.7%) | 678 (95.4%) | <0.001 | <0.001 | <0.001 | <0.001 |
| mbp<65 | 504 (10.1%) | 326 (7.71%) | 33 (60.0%) | 145 (20.4%) | <0.001 | <0.001 | <0.001 | <0.001 |
| CKD | 870 (17.4%) | 713 (16.9%) | 17 (30.9%) | 140 (19.7%) | 0.006 | 0.031 | 0.074 | 0.074 |
| IV | 155 [48.4;724] | 120 [20.9;342] | 583 [236;1156] | 3166 [2393;4063] | 0.000 | <0.001 | 0.000 | <0.001 |
