# Supplementary material for: An Easy and Quick Risk-Stratified Early Forewarning Model for Septic Shock in the Intensive Care Unit: Development, Validation, and Interpretation Study
Source: J Med Internet Res. 2025 Feb 6;27:e58779. doi: 10.2196/58779 (PMC11843061; doi:10.2196/58779)
Supplement: Multimedia Appendix 9 [file jmir_v27i1e58779_app9.docx]

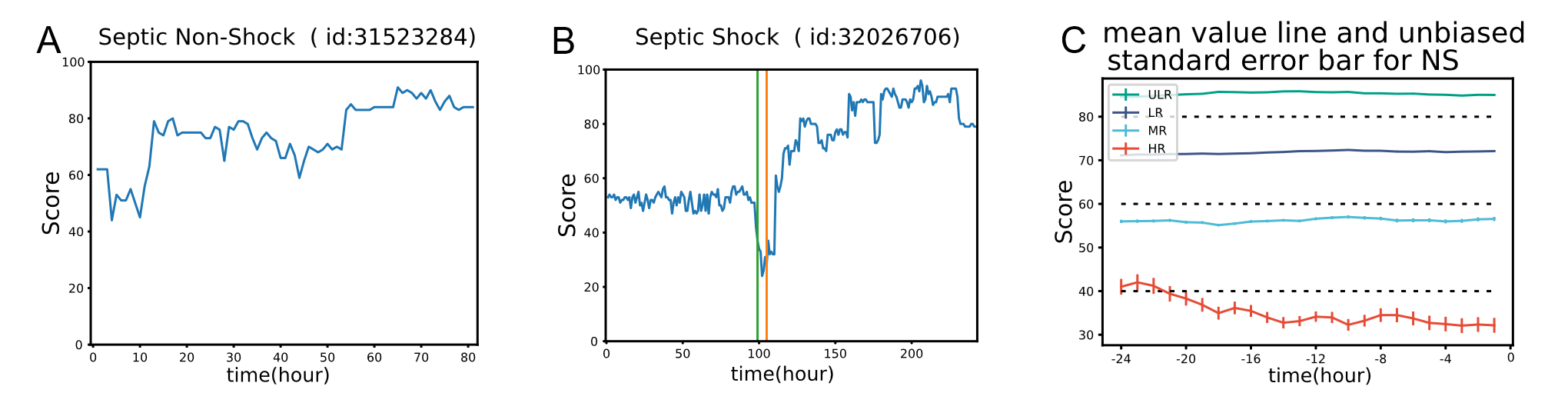


# Multimedia Appendix 9. The changing trends in risk across the whole intensive care unit (ICU) process. The changing trends in risk across the whole ICU process for two single patients: NS patient (A) and SS patient (B). The green line represents 6 hours before the onset of SS, and the orange line represents the onset time of SS. (C) Mean value line and unbiased standard error bar for NS patients. Risk groups derived by SORP 12 hours before discharge and the change in risk over time for each group.
