# Supplementary material for: An Easy and Quick Risk-Stratified Early Forewarning Model for Septic Shock in the Intensive Care Unit: Development, Validation, and Interpretation Study
Source: J Med Internet Res. 2025 Feb 6;27:e58779. doi: 10.2196/58779 (PMC11843061; doi:10.2196/58779)
Supplement: Multimedia Appendix 10 [file jmir_v27i1e58779_app10.docx]

# Multimedia Appendix 10. Medical Information Mart for Intensive Care-IV (MIMIC-IV) data for the invasive operation distribution and the significance of septic shock risk groups.

|  | [ALL]  N=711 | SS_O  N=646 | SS_LR  N=65 | OR | p.value |
| --- | --- | --- | --- | --- | --- |
| Invasive Ventilation: | 330 (46.4%) | 292 (45.2%) | 38 (58.5%) | 1.70 [1.02;2.89] | 0.043 |
| Dialysis Catheter: | 195 (27.4%) | 175 (27.1%) | 20 (30.8%) | 1.20 [0.67;2.07] | 0.524 |
| Arterial Line: | 380 (53.4%) | 343 (53.1%) | 37 (56.9%) | 1.17 [0.70;1.97] | 0.560 |
| Dialysis - CRRT: | 107 (15.0%) | 92 (14.2%) | 15 (23.1%) | 1.82 [0.95;3.31] | 0.071 |
