# Supplementary material for: An Easy and Quick Risk-Stratified Early Forewarning Model for Septic Shock in the Intensive Care Unit: Development, Validation, and Interpretation Study
Source: J Med Internet Res. 2025 Feb 6;27:e58779. doi: 10.2196/58779 (PMC11843061; doi:10.2196/58779)
Supplement: Multimedia Appendix 11 [file jmir_v27i1e58779_app11.docx]

# Multimedia Appendix 11. Medical Information Mart for Intensive Care-IV (MIMIC-IV) data for the invasive operation distribution and the significance of low-risk groups.

|  | [ALL]  N=2540 | NS_LR  N=2475 | SS_LR  N=65 | OR | p. value |
| --- | --- | --- | --- | --- | --- |
| Invasive Ventilation | 559 (22.0%) | 521 (21.1%) | 38 (58.5%) | 5.26 [3.19;8.80] | <0.001 |
| Dialysis Catheter | 198 (7.80%) | 178 (7.19%) | 20 (30.8%) | 5.75 [3.25;9.84] | <0.001 |
| Arterial Line | 783 (30.8%) | 746 (30.1%) | 37 (56.9%) | 3.06 [1.86;5.08] | <0.001 |
| Dialysis - CRRT | 44 (1.73%) | 29 (1.17%) | 15 (23.1%) | 25.3 [12.4;49.7] | <0.001 |
