# Supplementary material for: An Easy and Quick Risk-Stratified Early Forewarning Model for Septic Shock in the Intensive Care Unit: Development, Validation, and Interpretation Study
Source: J Med Internet Res. 2025 Feb 6;27:e58779. doi: 10.2196/58779 (PMC11843061; doi:10.2196/58779)
Supplement: Multimedia Appendix 12 [file jmir_v27i1e58779_app12.docx]

# Multimedia Appendix 12. eICU Collaborative Research Database (eICU) data for the rate of patients with septic shock in each risk group.

| group | septic shock | septic non-shock | total | rate |
| --- | --- | --- | --- | --- |
| high_risk | 97 | 96 | 193 | 0.5026 |
| medium_risk | 183 | 370 | 553 | 0.3309 |
| low_risk | 175 | 1317 | 1492 | 0.1173 |
| ultra_low_risk | 25 | 548 | 573 | 0.0436 |
