# Supplementary material for: An Easy and Quick Risk-Stratified Early Forewarning Model for Septic Shock in the Intensive Care Unit: Development, Validation, and Interpretation Study
Source: J Med Internet Res. 2025 Feb 6;27:e58779. doi: 10.2196/58779 (PMC11843061; doi:10.2196/58779)
Supplement: Multimedia Appendix 13 [file jmir_v27i1e58779_app13.docx]

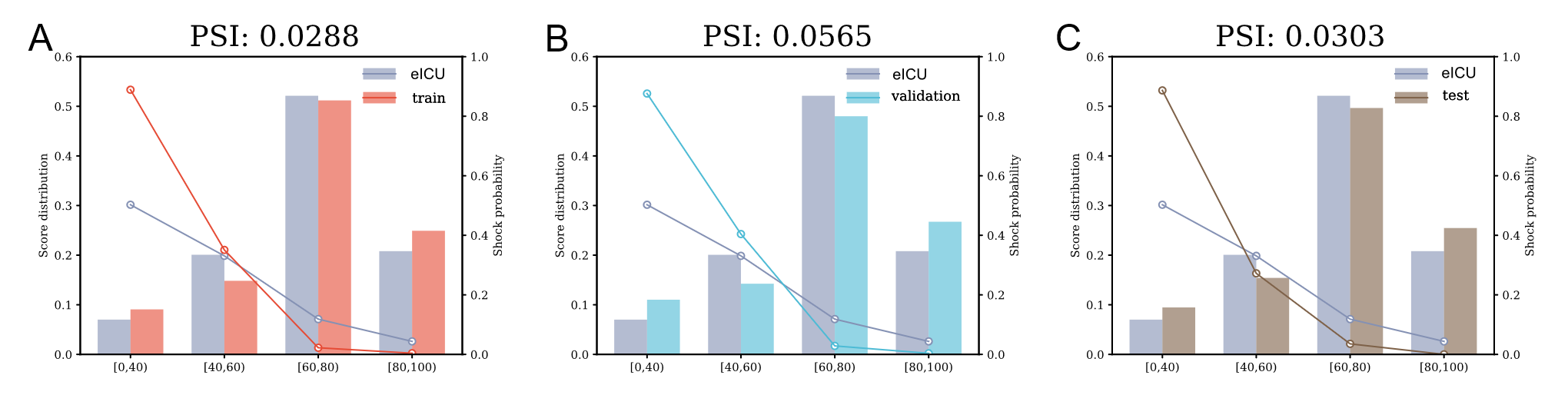


# Multimedia Appendix 13. Distribution stability of septic shock between the eICU Collaborative Research Database (eICU) and Medical Information Mart for Intensive Care-IV (MIMIC-IV) datasets. (A) for eICU and train dataset. (B) for eICU and validation dataset. (C) for eICU and test dataset.
