# Supplementary material for: An Easy and Quick Risk-Stratified Early Forewarning Model for Septic Shock in the Intensive Care Unit: Development, Validation, and Interpretation Study
Source: J Med Internet Res. 2025 Feb 6;27:e58779. doi: 10.2196/58779 (PMC11843061; doi:10.2196/58779)
Supplement: Multimedia Appendix 14 [file jmir_v27i1e58779_app14.docx]

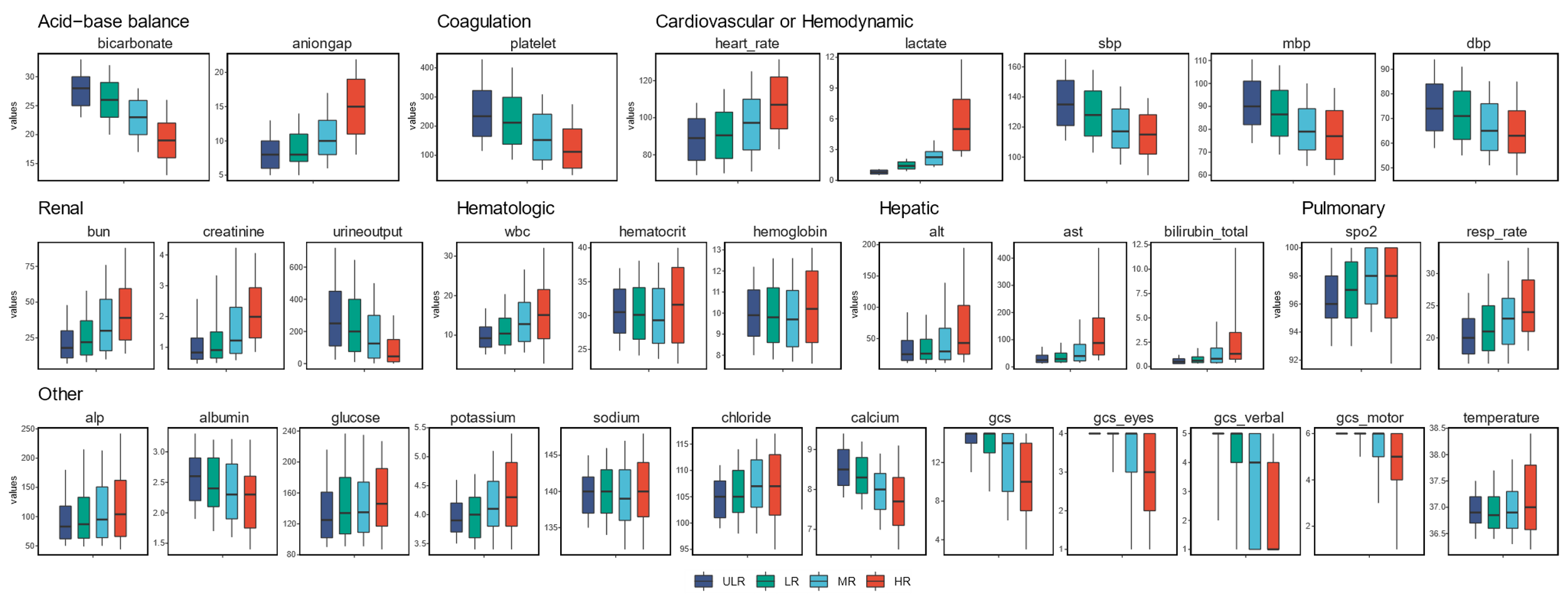


# Multimedia Appendix 14. Boxplots showing change trends for each clinical feature by risk groups in the eICU Collaborative Research Database (eICU) data. The y-axis shows the standardized value for each clinical feature.
