# Supplementary material for: An Easy and Quick Risk-Stratified Early Forewarning Model for Septic Shock in the Intensive Care Unit: Development, Validation, and Interpretation Study
Source: J Med Internet Res. 2025 Feb 6;27:e58779. doi: 10.2196/58779 (PMC11843061; doi:10.2196/58779)
Supplement: Multimedia Appendix 15 [file jmir_v27i1e58779_app15.docx]

# Multimedia Appendix 15. eICU Collaborative Research Database (eICU) data for the invasive operation distribution and the significance of low-risk groups.

|  | [ALL]  N=1332 | NS_LR  N=1174 | SS_LR  N=158 | OR | p.value |
| --- | --- | --- | --- | --- | --- |
| Mechanical Ventilation | 93 (6.98%) | 17 (1.45%) | 76 (48.1%) | 62.1 [35.9;114] | <0.001 |
| Dialysis-CRRT | 9 (0.68%) | 1 (0.09%) | 8 (5.06%) | 55.4 [9.85;1398] | <0.001 |
| Dialysis-Hemodialysis | 22 (1.65%) | 4 (0.34%) | 18 (11.4%) | 36.3 [13.2;130] | <0.001 |
