# Supplementary material for: An Easy and Quick Risk-Stratified Early Forewarning Model for Septic Shock in the Intensive Care Unit: Development, Validation, and Interpretation Study
Source: J Med Internet Res. 2025 Feb 6;27:e58779. doi: 10.2196/58779 (PMC11843061; doi:10.2196/58779)
Supplement: Multimedia Appendix 16 [file jmir_v27i1e58779_app16.docx]

# Multimedia Appendix 16. eICU Collaborative Research Database (eICU) data for the invasive operation distribution and the significance of septic shock risk groups.

|  | [ALL]  N=416 | SS_O  N=258 | SS_LR  N=158 | OR | p. value |
| --- | --- | --- | --- | --- | --- |
| Mechanical Ventilation | 196 (47.1%) | 120 (46.5%) | 76 (48.1%) | 1.07 [0.72;1.59] | 0.754 |
| Dialysis-CRRT | 26 (6.25%) | 18 (6.98%) | 8 (5.06%) | 0.72 [0.29;1.66] | 0.448 |
| Dialysis-Hemodialysis | 35 (8.41%) | 17 (6.59%) | 18 (11.4%) | 1.82 [0.90;3.69] | 0.095 |
