# Supplementary material for: An Easy and Quick Risk-Stratified Early Forewarning Model for Septic Shock in the Intensive Care Unit: Development, Validation, and Interpretation Study
Source: J Med Internet Res. 2025 Feb 6;27:e58779. doi: 10.2196/58779 (PMC11843061; doi:10.2196/58779)
Supplement: Multimedia Appendix 17 [file jmir_v27i1e58779_app17.docx]

# Multimedia Appendix 17. eICU Collaborative Research Database (eICU) data for the clinical event distribution and the significance of risk groups.

|  | [ALL]  N=2554 | NS_O  N=2030 | NS_HR N=84 | SS  N=440 | p.overall | p.NS_O vs NS_HR | p.NS_O vs SS | p.NS_HR vs SS |
| --- | --- | --- | --- | --- | --- | --- | --- | --- |
| vaso | 316 (12.4%) | 26 (1.28%) | 16 (19.0%) | 274 (62.3%) | <0.001 | <0.001 | <0.001 | <0.001 |
| mbp<65 | 170 (6.66%) | 72 (3.55%) | 30 (35.7%) | 68 (15.5%) | <0.001 | <0.001 | <0.001 | <0.001 |
