# Supplementary material for: An Easy and Quick Risk-Stratified Early Forewarning Model for Septic Shock in the Intensive Care Unit: Development, Validation, and Interpretation Study
Source: J Med Internet Res. 2025 Feb 6;27:e58779. doi: 10.2196/58779 (PMC11843061; doi:10.2196/58779)
Supplement: Multimedia Appendix 18 [file jmir_v27i1e58779_app18.docx]

# Multimedia Appendix 18. Current studies for predicting the onset of septic shock.

| Auth | Model name | AUC | HBO Median (IQR) | Features No. | Features |
| --- | --- | --- | --- | --- | --- |
| Henry et al.[1] | TREWScore | 0.83 | 28.2  (10.6, 94.2) | 54 | HR; SBP; Shock index* = hr/sbp; Urine 6hr; GCS; RR; Time since first antibiotics*; Chronic liver disease and cirrhosis; Cardiac surgery patient; Immunocompromised; Hematological malignancy; Chronic heart failure; Chronic organ insufficiency; Diabetes; Metastatic carcinoma; FiO2; BUN/CR; Arterial pH; PaO2; BUN; WBC; Platelets; Renal SOFA; Neurologic SOFA; Hepatic SOFA; SIRS…(Lack of complete list) |
| Fagerström et al.[2] | LiSep LSTM | 0.83 | 40  (20, 135) | 24 | Age; Systolic Blood Pressure; Diastolic Blood Pressure; Mean Arterial Blood Pressure; Glasgow Coma Scale; Heart Rate; Fluid Input; Urine Output; Respiratory Rate; Antibiotics; Partial Carbon Dioxide Pressure; Partial Oxygen Pressure; Fraction of Inspired Oxygen; Oxygen Saturation; Bicarbonate Level; Blood Urea Nitrogen; Arterial pH; Creatinine; Potassium; Platelet Count; Hematocrit; Hemoglobin; White Blood Cell Count; Riker Sedation-Agitation Scale |
| Liu et al.[3] | pre-shock RNN | 0.93 | 7.0  (N/A) | 10 | GCS; HR; Respiratory Rate; Lactate; PaO2; FiO2; Cardiovascular SOFA; Kidney SOFA; Respiratory SOFA; Coagulatory SOFA |
| Mollura et al.[4] | Logistic regression | 0.93 | 0.25  (N/A) | waveforms | ECG and ABP waveforms |
| **Our** | SORP | 0.95 | 13  (6, 26) | 15 | heart_rate; resp_rate; temperature; spo2; dbp; mbp; sbp; lactate; bicarbonate; glucose; hematocrit; sodium; potassium; hemoglobin; calcium; |

Note: Only vital signs and blood gas data, which are very easy to obtain in the ICU, were needed, and a quick blood gas report can be obtained in 5-10 minutes. Other models have many additional tests, such as routine blood examination, SOFA score calculation, and urine output at a certain time. This leads to the need for long-term data collection. HBO, hours before septic shock onset.

**Reference**

1. Henry KE, Hager DN, Pronovost PJ, Saria S. A targeted real-time early warning score (TREWScore) for septic shock. Sci Transl Med. 2015 Aug 5;7(299):299ra122. PMID: 26246167. doi: 10.1126/scitranslmed.aab3719.

2. Fagerstrom J, Bang M, Wilhelms D, Chew MS. LiSep LSTM: A Machine Learning Algorithm for Early Detection of Septic Shock. Sci Rep. 2019 Oct 22;9(1):15132. PMID: 31641162. doi: 10.1038/s41598-019-51219-4.

3. Liu R, Greenstein JL, Granite SJ, Fackler JC, Bembea MM, Sarma SV, et al. Data-driven discovery of a novel sepsis pre-shock state predicts impending septic shock in the ICU. Sci Rep. 2019 Apr 16;9(1):6145. PMID: 30992534. doi: 10.1038/s41598-019-42637-5.

4. Mollura M, Romano S, Mantoan G, Lehman L-w, Barbieri R, editors. Prediction of Septic Shock Onset in ICU by Instantaneous Monitoring of Vital Signs. 2020 42nd Annual International Conference of the IEEE Engineering in Medicine & Biology Society (EMBC); 2020: IEEE.
