# Supplementary material for: An Easy and Quick Risk-Stratified Early Forewarning Model for Septic Shock in the Intensive Care Unit: Development, Validation, and Interpretation Study
Source: J Med Internet Res. 2025 Feb 6;27:e58779. doi: 10.2196/58779 (PMC11843061; doi:10.2196/58779)
Supplement: Multimedia Appendix 19 [file jmir_v27i1e58779_app19.docx]

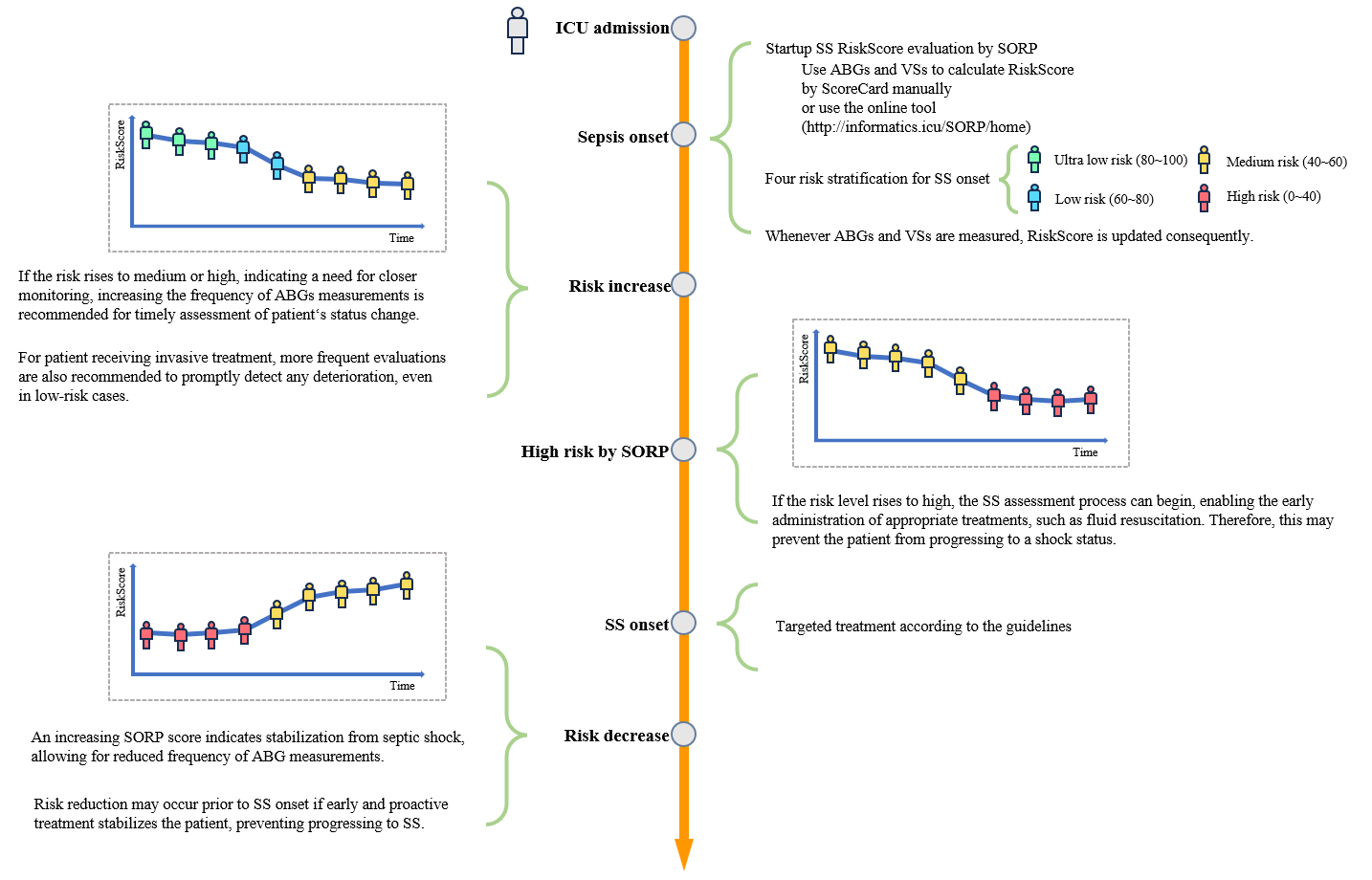


# Multimedia Appendix 19. Schematic diagram for auxiliary monitoring of the Septic Shock Risk Predictor (SORP) in clinical practice. ICU: Intensive Care Unit; SS: Septic Shock; ABGs: Arterial Blood Gases; VSs: Vital Signs.
